# Isovitexin Inhibits Ginkgolic Acids-Induced Inflammation Through Downregulating SHP2 Activation

**DOI:** 10.3389/fphar.2021.630320

**Published:** 2021-08-11

**Authors:** Yiwei Zhang, Zhipeng Qi, Wenjie Wang, Lei Wang, Fuliang Cao, Linguo Zhao, Xianying Fang

**Affiliations:** ^1^ College of Chemical Engineering, Nanjing Forestry University, Nanjing, China; ^2^ Co-Innovation Center for Sustainable Forestry in Southern China, Nanjing Forestry University, Nanjing, China

**Keywords:** isovitexin, ginkgolic acids, dermatitis, inflammation, SHP2, *Celtis sinensis* Pers. leaf

## Abstract

It has been reported that *Celtis sinensis* Pers. is employed as a folk medicine for the treatment of inflammatory diseases. But the mechanism supporting its use as anti-inflammatory remains unclear. To investigate the anti-inflammatory of *Celtis sinensis* Pers. ICR mice were provided *Celtis sinensis* Pers. leaf extract (CLE) at 100, 200 mg/kg after ginkgolic acids (GA) sensitization. Our data showed that CLE and the main flavonoid isovitexin in CLE could ameliorate GA-induced contact dermatitis in mice. Ear swelling, inflammatory cell infiltration and splenomegaly were inhibited significantly by isovitexin, while the weight loss of mice in the isovitexin-treated group was much better than that in the dexamethasone-treated group (positive control drug). It has been reported in previous research that GA-induced inflammation is closely related to the T cell response. Therefore, T cells were the focus of the anti-inflammatory effect of isovitexin in this paper. The *in vivo* results showed that isovitexin (10, 20 mg/kg) inhibited the expression of proinflammatory cytokines (TNF-α, IFN-γ, IL-2 and IL-17A) in lymph nodes, inhibited the secretion of cytokines into the serum from mice with contact dermatitis and promoted the expression of apoptosis-related proteins. *In vitro*, isovitexin also induced apoptosis and inhibited proinflammatory cytokine expression in Con A-activated T cells. Further study showed that the MAPK and STAT signaling pathways and the phosphorylation of SHP2 were inhibited by isovitexin. Both molecular docking and biological experiments indicated that SHP2 may be an anti-inflammatory target of isovitexin in T cells. Taken together, isovitexin can serve as a potential natural agent for the treatment or prevention of GA-induced inflammatory problems.

## Introduction

Ginkgo seeds has been used as a nutritious food for thousands of years, and a variety of medicinal effects have been attributed to the ginkgo seeds. The main active pharmaceutical ingredients in *Ginkgo biloba* include oxyglycoside flavonoids, terpene trilactones, proanthocyanidins and so on (Zhou et al., 2018; Omidkhoda et al., 2019). However, food poisoning by Ginkgo seeds has been reported in Japan and China, which presents as frequent vomiting and generalized convulsions (Miwa et al., 2001). Ginkgolic acids (GA), the alkylphenol constituents in ginkgo seeds, have been considered one of the potential toxic components in *Ginkgo biloba*. The functional disorders are probably due to GA (Qian et al., 2017). It has also been reported that contact allergic dermatitis (ACD) can be induced by ginkgolic acids when people contact ginkgo leaf during the picking of Ginkgo seeds (Hotta et al., 2013; Mei et al., 2017). *Ginkgo biloba* leaf exhibit useful applications in health, food and dietary supplements but are also controversial in the application of some aspects because of the existence of GA. Under some conditions, GA can be separated by organic solvent extraction or column chromatography (Van Beek and Montoro, 2009). However, for raw material products such as Ginkgo seeds and ginkgo tea, the GA removal process cannot be performed (Fransen et al., 2010; Fang et al., 2019). Other methods, such as detoxification by compatibility, need to be taken to solve the GA-caused toxicity problems in the application of Ginkgo seeds and ginkgo tea.

GA, a derivative of alkyl-substituted salicylic acid, is similar to the chemical structure of urushiol and differs by carbon number and unsaturation, classified as C13:0, C15:1, C15:0, and C17:1 (Wang et al., 2014). Allergies were caused by paint occur frequently in our daily lives. As an allergen from paint, urushiol can cause intense, persistent itch, skin rashes and a burning sensation in severe cases (Boelman, 2010). The clinical manifestations of GA-induced ACD are similar to urushiol-induced ACD, followed by the appearance of erythematous edematous plaques and papulovesicles accompanied by intense pruritus on the forearms in severe cases (Han et al., 2016). These inflammatory reactions can lead to a series of problems, such as skin injury, immune liver injury, gastrointestinal inflammatory injury and so on. With an important role in the immune response, T cells appear to proliferate and differentiate. However, overdeveloped T cells are associated with cutaneous allergic and inflammatory responses, which exacerbate skin inflammation, tissue injury and other immunopathies. In a previous study, reports suggested that the mechanism of GA-induced ACD may be consistent with urushiol-induced ACD. Numerous experimental animal models have validated GA as a hapten with the ability to activate T cells against innocuous or autoantigens and induce type IV allergic reactions (Ude et al., 2013). A study has shown that T cells in rats sensitized with GA are differentiated into CD4^+^ T cells, suggesting that GA may act as an allergen to enhance body sensitivity, induce T cell division and proliferation and enhance the cellular immune response.

*Celtis sinensis Pers.* has been used as a traditional herbal remedy for thousands of years in China. It uses to treat urushiol-induced dermatitis. There is a folk tale, from the Maolan karst forest, that if someone contacted poison ivy inadvertently, he could be treated by putting the leaf of *Celtis sinensis Pers.* into his mouth. Isovitexin, the most abundant flavonoid in the leaf of *Celtis sinensis* (Zehrmann et al., 2010), is a widely found natural carbon glycoside flavonoid*.* Isovitexin has various pharmacological activities, such as antineoplastic (Cao et al., 2019), antioxidant (Liu et al., 2020a), and neurological protective effects (Liu et al., 2020b). This compound has also shown anti-inflammatory efficacy against human diseases (Fransen et al., 2010; Fang et al., 2019). It has been reported that isovitexin inhibits the MAPK and NF-κB pathways in macrophages in acute lung injury (Fransen et al., 2010; Fang et al., 2019). In lymphocytes, a number of ACD-associated cytokines are dependent on JAK-STAT signaling, and antigen presentation is dependent on MAPK signaling for the effects on cellular transcription and activation. In this study, a contact-hypersensitivity mouse model induced by GA was established to reveal the mechanisms underlying the anti-inflammatory effect of isovitexin. In summary, this study provides supporting data for isovitexin ameliorating GA-induced allergic contact dermatitis.

## Materials and Methods

### Reagents

Isovitexin (CAS No. 38953-85-4, purity: 98.01%) was obtained from Mansite Biotechnology, Chengdu, China) was dissolved in 100% DMSO at a concentration of 100 mM as a stock solution and diluted test concentration with culture medium before each experiment. The concentration of final DMSO did not extend 0.1% throughout the trail. Ginkgolic acids were purchased from Jingzhu Medical Technology (Nanjing, China). The Annexin V-FITC/PI apoptosis kit and ELISA kits for murine (TNF-α, IFN-γ, IL-2, IL-17A) were purchased from MultiSciences (Lianke) Biotech Co., Ltd. (Hangzhou, China). SHP099, the SHP2 inhibitor, purchased from Selleckchem. Concanavalin A (Con A), MTT, Freund’s Adjuvant Complete (FAC) and Dexamethasone (Dex) were purchased from Sigma-Aldrich (St Louis, MO). Antibodies against phospho-STAT3 (Tyr705), phospho-AKT (Ser473), Anti-ERK, phospho-P38 (Thr180/Tyr182), phospho-JNK (Thr183/Tyr185), phospho-ERK1/2 (Thr 202/Tyr 204), Caspase-3, Caspase-8, Cleaved Caspase-3 (Asp175), Cleaved Caspase-8 (Asp387), Cleaved PARP (Asp214) were purchased from Cell Signal Technology (Beverly, MA). Antibodies against phospho-SHP2 (Tyr 542) was purchased from Abcam (Cambridge, United Kingdom). Anti-β-actin, Anti-AKT, Anti-PARP, Anti-P38, Anti-JNK, Anti-AKT, Anti-STAT6 was purchased from Proteintech Group (Wuhan, China). Anti-STAT3, Anti-SHP2 was purchased from Santa cruz Biotechnology.

### Animal

Female ICR mice and BALB/c mice, 18–23 g, 6–8 weeks old, were obtained from the Experimental Animal Center of Yangzhou University (Yangzhou, China). The mice were maintained in plastic cages with free access to eat food and drink water, temperature kept at 21 ± 2°C and kept on a 12 h light/dark cycle. Animal welfare and experimental procedures were subjected to the Guide for the Care and Use of Laboratory Animals (National Institutes of Health, United States), and the study protocol was approved by the Animal Care and Protection Committee of Nanjing University-Gulou Hospital (SYXK 2004-0013). The authors confirmed that all animals received human care and all animal experiments were performed in accordance with the relevant guidelines and regulations. All the authors complied with the ARRIVE guidelines experiments.

**FIGURE 1:**
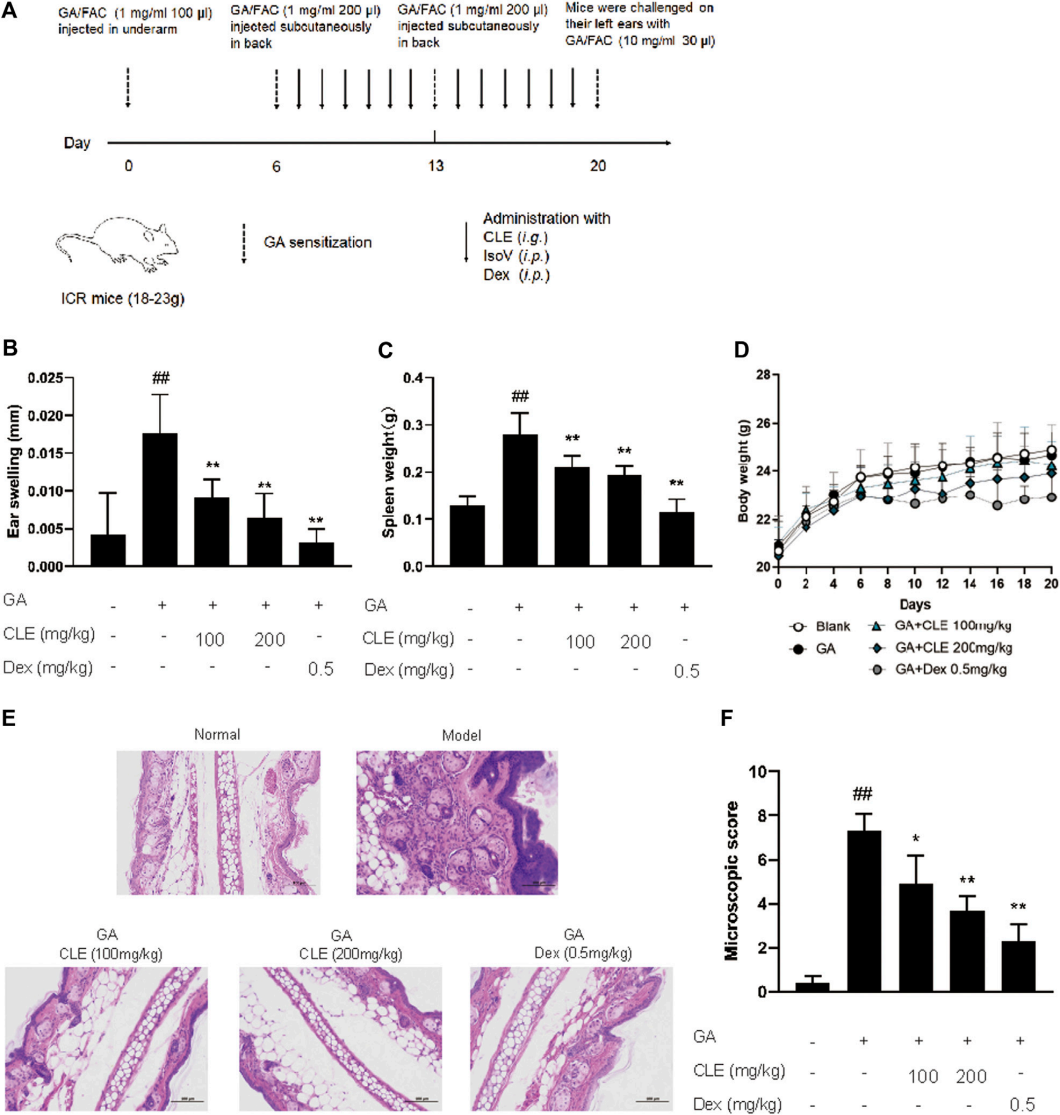
Isovitexin decreases the susceptibility of GA-induced contact dermatitis in mice. In the mouse model of GA-induced contact dermatitis, mice were given isovitexin, a main flavonoid compound from CEL, at 10 and 20 mg/kg respectively. **(A)** Chemical structural of isovitexin. **(B)** The ear swelling was measured at day 20. **(C)** Spleen weight was measured at day 20. **(D)** The body weight changes of mice in different groups. **(E)** Hematoxylin and eosin stain (original magnification ×200) of ear tissue sections. Bars, 100 µm. **(F)** Ear histological scoring. IsoV, isovitexin. Data represented means ± SEM of eight mice. ^##^ *p* < 0.01 vs. vehicle group, *p < 0.05, **p < 0.01 vs. model group.

Experimental design for GA-induced contact hypersensitivity is shown in Figure 1A. On the first day (day 0), female ICR mice were sensitized with FAC emulsified GA (GA/FAC, 1 mg/ml, 100 μl) in the right flank by subcutaneous injection. On Day 6 and day 13, mice were sensitized with FAC emulsified GA (GA/FAC, 1 mg/ml, 200 μl) in the back by subcutaneous injection of multipoints. On the last day (day 20), mice were challenged on their left ears with GA/FAC (10 mg/ml, 30 μl). 24 h after the challenge, the ear thickness was measured with a digimatic micrometer. Ears’ swelling was evaluated by the thickness difference between the left and right. Mice in normal group were normally sensitized with FAC and challenged with olive oil without GA. CLE (100, 200 mg/kg, intragastrically) or isovitexin (10, 20 mg/kg, intraperitoneally) or dexamethasone (0.5 mg/kg, intraperitoneally) was administered once a day from day 6 to day 20. Mice in the normal group and model group were given saline as control.

### Cell Culture and Proliferation Assay

T cells, isolated from female Balb/c mice Lymph nodes, were cultured in 96-well plates in 1,640 medium at the density of 5 × 10^6^ cells/well and stimulated with 5 μg/ml of Con A for 48 h under a humidified 5% (v/v) CO_2_ atmosphere at 37°C. MTT with concentration of 4 mg/ml dissolves in PBS, 20 μl of MTT was added in each well 4 h before the end of incubation. Then culture media was removed and 200 μl DMSO was added to dissolve the crystals. Measuring the absorbance value at 540 nm.

### Plant Material Extraction of Celtis Leaf

The plant name, *Celtis sinensis Pers.*, were confirmed with the Medicinal Plant Names Services (http://mpns.kew.org). Celtis leaves were collected from Guizhou, China, and were authenticated as *C. sinensis* based on the morphological characteristics by the international Cultivar Registration Center (Nanjing Forestry University). A voucher specimen (voucher number GZCS200503) of this plant was deposited at 2,304 laboratory, college of chemical engineering, Nanjing Forestry University, China.

The method of extraction followed with few modifications as described by Lee et al. (2018). The air-dried leaf of Celtis sinensis (5 kg) were ground in a cutting mill and soaked with ethyl alcohol (25L′3, for 1.5 h each) at 70°C. After the crude extract was dried under reduced pressure, a crude dark green residue was suspended in water with freeze-dried preservation.

### Quantitative RT-PCR

Total RNA was extracted from popliteal fossa lymph nodes and reverse transcribed to cDNA. Quantitative PCR was performed with the ABI Prism 7,000 sequence detection system (Applied Biosystems, Foster City, CA)using SYBR Green I dye (Biotium, Inc.) Threshold cycle numbers were obtained using ABI Prism 7000 SDS software version 1.0. PCR cycling conditions were as follows: one cycle of 94 8C for 5 min followed by 40 cycles of 94 8C for 30 s, 58 8C for 30 s, and 72 8C for 45 s. The primer sequences used were as follows:

tnf-α forward 5′-CCC​TCA​CAC​TCA​GAT​CAT​CTT​CT-3′,

tnf-α reverse 5′-GCT​ACG​ACG​TGG​GCT​ACA​G-3′;

ifn-γ forward 5′-GCC​ACG​GCA​CAG​TCA​TTG​A-3′,

ifn-γ reverse 5′-TGC​TGA​TGG​CCT​GAT​TGT​CTT-3′;

il-2 forward 5′-GTG​CTC​CTT​GTC​AAC​AGC​G-3′,

il-2 reverse 5′-GGG​GAG​TTT​CAG​GTT​CCT​GTA-3′;

il-17a forward 5′-TTT​AAC​TCC​CTT​GGC​GCA​AAA-3′,

il-17a reverse 5′-CTT​TCC​CTC​CGC​ATT​GAC​AC-3′;

β-actin forward 5′-GTG​ACG​TTG​ACA​TCC​GTA​AAG​A-3′,

β-actin reverse 5′-GCC​GGA​CTC​ATC​GTA​CTC​C-3′.

Relative message RNA (mRNA) expression was calculated as a ratio to actin.

### Cytokine Assay

Cytokine levels were measured using ELISA kits from MultiSciences (Lianke) Biotech Co., Ltd. (Hangzhou, China) according to the manufacturer’s instructions.

### Western Blot Analysis

Cells isolated from lymph node were cultured in 6-well plates at a density of 1 × 10^7^ cells/well in RPMI1640 medium and stimulated with Con A (5 μg/ml). Proteins lyzed from cultured cells were separated by SDS-PAGE and electrophoreticically transferred onto PVDF membranes (Millipore, Bedford, MA). After treatment with blocking buffer in 5% BSA at RT for 1 h, membranes were incubated with primary antibodies at 4°C overnight, and the secondary antibody incubation at RT for 2 h. Antibody reactivity was detected by using ECL luminescence reagent (Tanon, Shanghai).

### Histological Analysis

The 5 mm thickness of ear sections were obtained by formalin-fixed, paraffin-embedded and stained with hematoxylin and eosin. Histological parameter was following as described before (Omidkhoda et al., 2019): the level of leukocyte infiltration and vascular congestion (Zhou et al., 2018); the erosion and anabrosis of epidermal cells (Miwa et al., 2001); affection of the other side of the ears (Luo et al., 2011). We scored each of the histological assessment on a scale of 1–4 and the higher score means more serious inflammation.

### Molecular Docking Analysis

The molecular docking analysis was conducted in Maestro v11.1 (Schrödinger, LLC) by the default protocols (Wang et al., 2020). We prepared the ligand isovitexin and SHP2 protein (Protein Data Bank ID:3o5x). The docking grid was generated based on the position of the tyrosine phosphatase SHP2 with the default protocol. Subsequently, glide docking was performed and induce-fit docking was conducted based on the results of glide docking.

### Statistical Analysis

All Data were represented means ± SEM from triplicate experiments performed in a parallel manner. Data were statistically compared and one-way analysis of variance ANOVA followed by Dunnett’s test between the vehicle group and multiple dose groups (Szczepanski et al., 2020). The level of significance was set at *p* < 0.05.

## Results

### *Celtis sinensis* Leaf Extract Protects Mice From Allergic Contact Dermatitis

*Celtis sinensis* is employed as a folk medicine for treating inflammation, skin infections and other diseases (Rojas-Bedolla et al., 2018). To study the possibility of using *Celtis sinensis* to treat inflammation in GA-induced allergic contact dermatitis, a mouse model of GA-induced hypersensitive contact dermatitis was established. We stimulated the popliteal lymph nodes and the left ear of ICR mice by GA (Figure 1A). Mice challenged by GA displayed definite inflammation, as indicated by splenomegaly and ear swelling, compared with the vehicle group.

*Celtis sinensis* leaf extract (investigational ingredient, CLE) and dexamethasone (positive control drug, Dex) were used to treat the degree of ear swelling. Notably, mice that received CLE exhibited significantly reduced susceptibility to GA-induced allergic contact dermatitis, as shown by the milder splenomegaly and ear swelling compared with the model group (Figures 1B,C). Indeed, CLE did not decrease the body weight of mice, but it appeared in mice that received dexamethasone (Figure 1D). Histopathologically, mice that received CLE displayed significantly alleviated leukocyte infiltration, epidermal anabrosis and affection damage compared with the vehicle group (Figures 1E,F). Collectively, intragastric instillation of CLE confers protection from GA-induced allergic contact dermatitis in ICR mice.

### Isovitexin, the Main Flavonoid in the Leaf of *Celtis sinensis*, Ameliorates GA-Induced Allergic Contact Dermatitis in Mice

**FIGURE 2:**
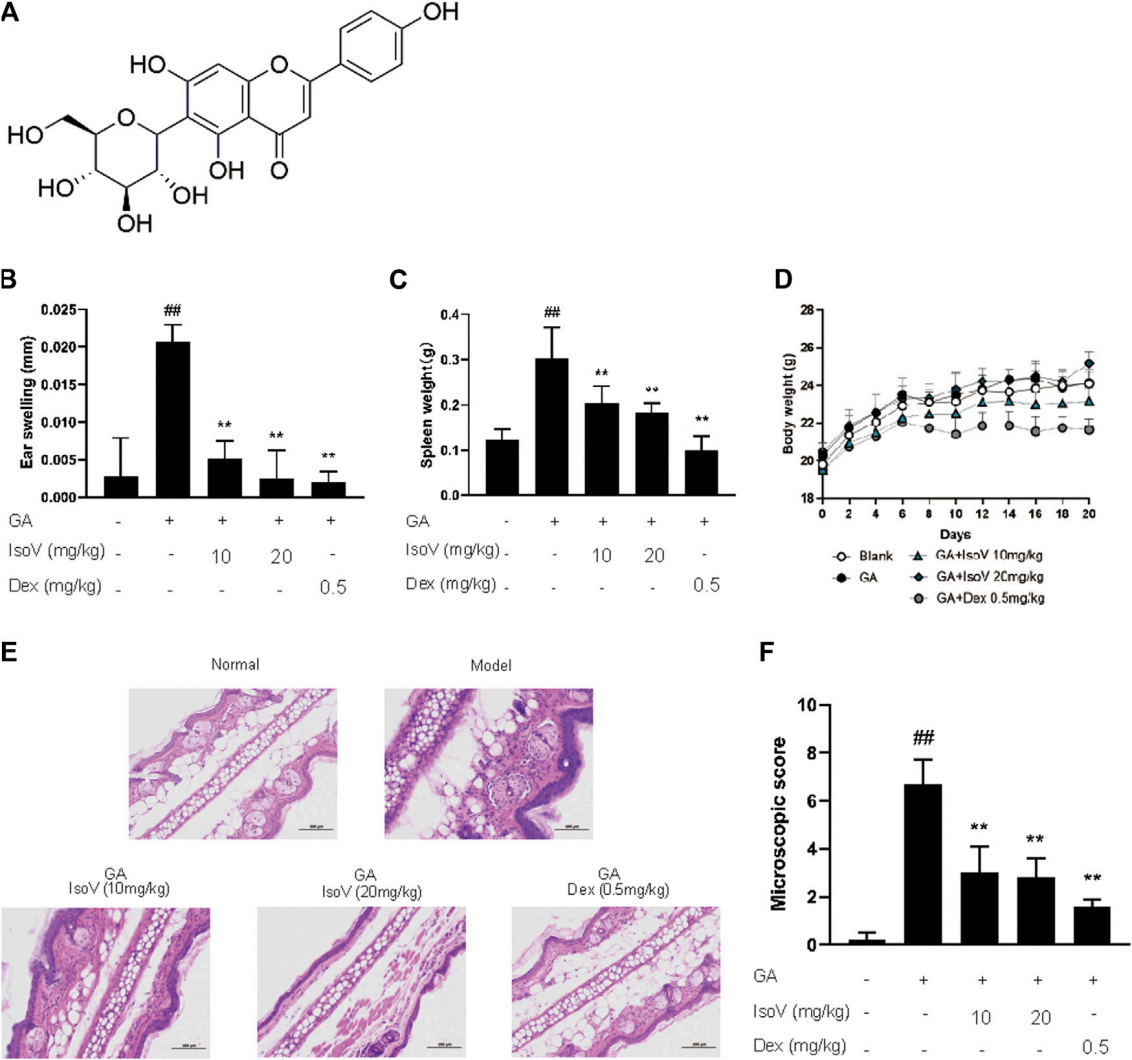
*Celtis sinensis* leaf Extract (CLE) ameliorates ginkgolic acids (GA)-induced contact dermatitis in mice. **(A)** The mouse model of GA-induced contact dermatitis, drugs were administered once a day from day 6 to day 20. **(B)** The mice were given CLE at 100 and 200 mg/kg respectively, and the ear swelling was measured 24 h after challenge at day 20. **(C)** Spleen weight was measured at day 20. **(D)** The body weight changes of mice in different groups. **(E)** Hematoxylin and eosin stain (original magnification ×200) of ear tissue sections. Bars, 100 µm. **(F)** Ear histological scoring. Data represent means ± SEM of eight mice. ^##^ *p* < 0.01 vs. vehicle group, *p < 0.05, **p < 0.01 vs. model group.

Since isovitexin (chemical structure is shown in Figure 2A) is the predominant flavone of *Celtis sinensis* (Ota et al., 2017), we further examined the possible role of isovitexin in dermal inflammation. Mice that received isovitexin exhibited ameliorative ear swelling and splenomegaly compared with the model group (Figures 2B,C). Meanwhile, consistent with the CLE experiments, isovitexin did not show body weight loss, suggesting that compared with dexamethasone, isovitexin displayed significant anti-inflammatory function without serious side effects (Figure 2D). Histopathologically, mice that received isovitexin displayed directly reduced leukocyte chemotaxis and ameliorated proinflammatory cytokine release (Figures 2E,F). Therefore, isovitexin, a natural flavone, exerts immunosuppressive effects in GA-induced allergic contact dermatitis.

### Isovitexin Inhibits T Cell-Mediated Inflammation in Mice With GA-Induced Allergic Contact Dermatitis

**FIGURE 3:**
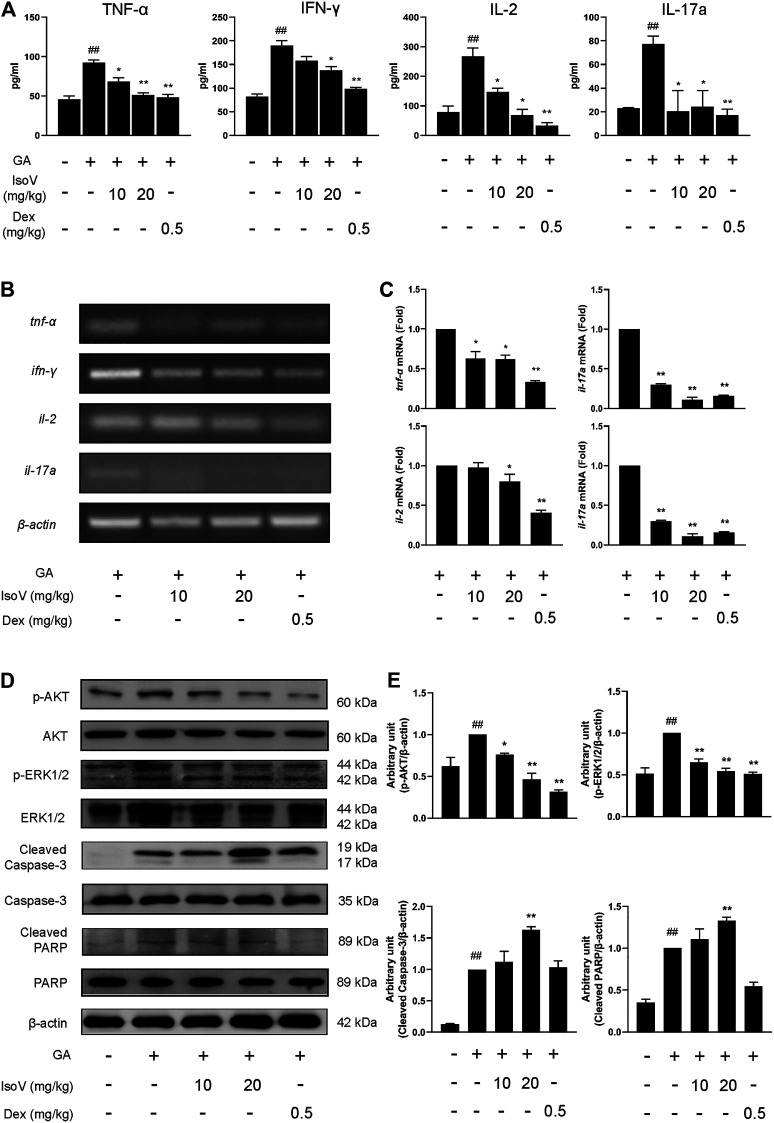
Isovitexin inhibits the expression of pro-inflammatory cytokines in lymph nodes and serum from mice with contact dermatitis and promotes the expression of apoptosis-related proteins. In the mouse model of GA-induced contact dermatitis, **(A)** the effect of isovitexin on the levels of pro-inflammatory cytokines in the serum was determined by ELISA. Data are representative of three independent experiments; **(B,C)** the effect of isovitexin on the inflammatory cytokines mRNA expression in lymph nodes was measured by RT-PCR, and the relative band density was analyzed using ImageJ; **(D,E)** the lymph node cells were harvested and lyzed, and the effect of isovitexin on the protein levels of p-AKT, Cleaved PARP, Cleaved Caspase-3, p-ERK1/2 were analyzed by western blotting, and the relative band density was analyzed using ImageJ. The grouping of gels were cropped from different parts of the different gels. Data represented means ± SEM of three independent time. ^##^ *p* < 0.01 vs. vehicle group, *p < 0.05, **p < 0.01 vs. model group.

Stimulation with GA also exacerbated the release of inflammatory cytokines (TNF-α, IFN-γ, IL-2, and IL-17A) in serum. The upregulation of these T cell-specific cytokines indicates that GA-induced allergic contact dermatitis mainly triggers inflammatory activation of T cells rather than macrophage-mediated innate inflammation. Anti-inflammatory effects of isovitexin were presumably attributed to a decrease in T cell-specific cytokines in serum (Figure 3A). We then evaluated the mRNA level of T cell-associated cytokines in popliteal lymph nodes. Compared with inflamed dermatitis mice, the contents of *tnf-*α*, ifn-*γ*, il-2,* and *il-17a* were significantly lower in mice that received isovitexin (Figures 3B,C). Moreover, we observed that proapoptotic proteins (cleaved caspase-3 and cleaved PARP) were activated and that proliferation proteins (phosphor-AKT and phospho-ERK1/2) were inhibited in isovitexin-treated T cells from allergic mice (Figures 3D,E). Taken together, these data clearly demonstrate that isovitexin may inhibit T cell-mediated inflammatory responses in dermatitis.

### Isovitexin Inhibits the Proliferation and Promotes Apoptosis of Con A-Activated T Cells *in vitro*

**FIGURE 4:**
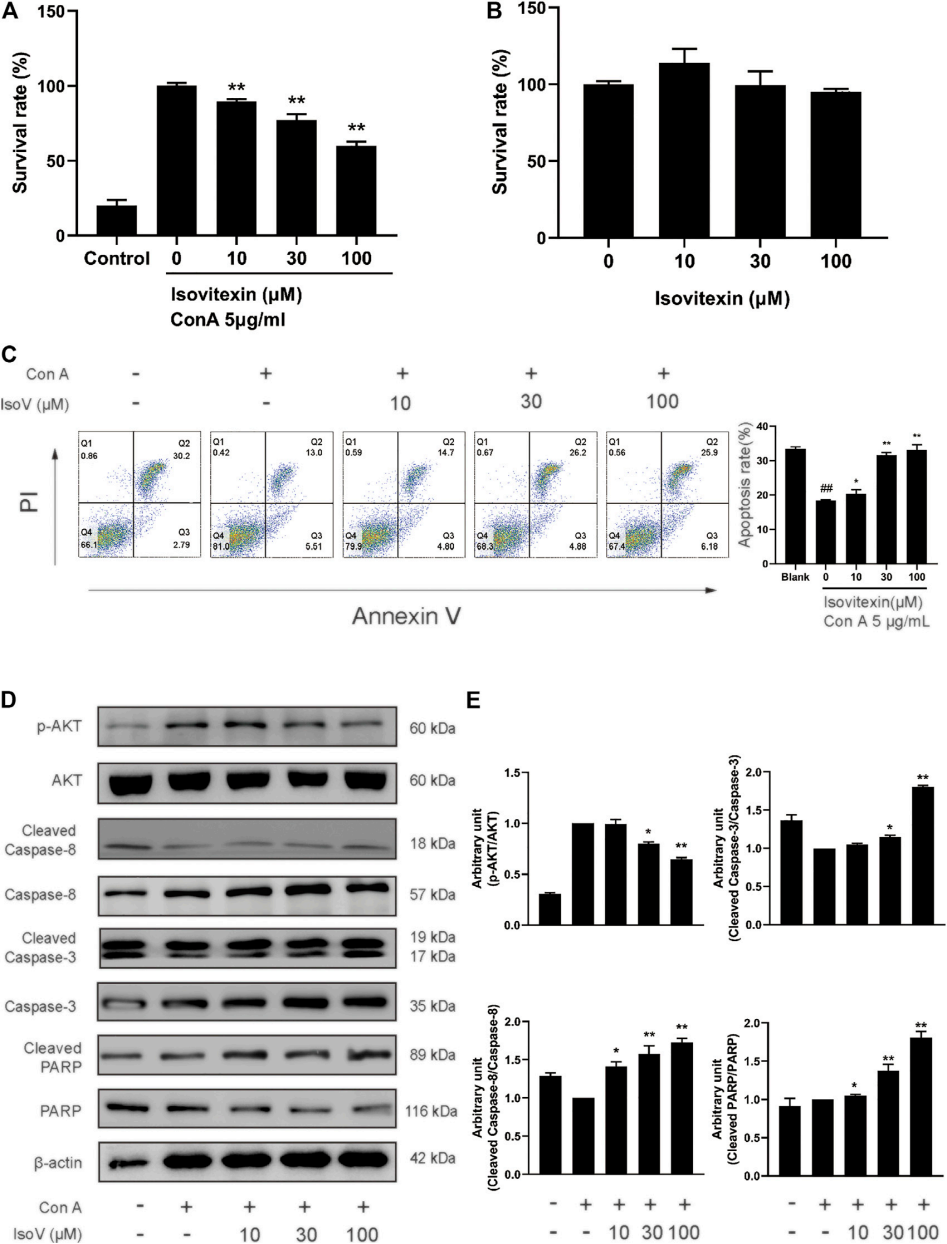
Isovitexin induces the apoptosis of Con A-activated T cells. **(A)** Con A-activated T cells were treated with different concentrations of isovitexin for 48 h, and the cell survival was determined by MTT assay. **(B)** T cells were treated with different concentrations of isovitexin for 24 h, and the cell survival was determined by MTT assay. Cells incubated without Con A and isovitexin were used as control. Con A-activated T cells were seeded in 12-well plates and incubated with 0, 10, 30 and 100 μM isovitexin for 24 h. **(C)** Cells were stained with Annexin V and PI, and the apoptosis of the cells was determined by flow cytometry assay of Annexin V/PI staining. Annexin V positive cells of three independent experiments were shown in column statistics. **(D,E)** Cells treated with different concentrations of isovitexin were harvested and lyzed. The protein levels of p-AKT, Cleaved caspase-3, Cleaved caspase-8, Cleaved PARP in T cells were measured by western blotting, and the relative band density was analyzed using ImageJ. The grouping of gels were cropped from different parts of the different gels. Data represented mean ± SEM of three independent experiments. ^##^ *p* < 0.01 vs. Blank group, *p < 0.05, **p < 0.01 vs. control (Con A-activated T cells).

The above findings prompted us to suppose that isovitexin diminished T cell proinflammatory activities in response to GA stimulation. Furthermore, we used Con A-activated T cells to explore the mechanisms underlying the antagonizing effects of isovitexin on metabolic disorders. Con A is a plant lectin that induces the mitogenic activity of T lymphocytes and increases the production of inflammatory cytokines such as IL-2, TNF-α and IFN-γ (Shinohara and Tsukimoto, 2017). The MTT assay was used to assess cell viability. As showing in Figure 4B, Con A (5 μg/ml) strongly promoted T cell proliferation. In addition, cell culture with the addition of 0–100 μM isovitexin was not toxic to naïve T cells, but it inhibited the proliferation of Con A-activated T cells (Figures 4A,B). On the other hand, isovitexin triggered apoptosis of Con A-activated T cells, as analyzed by the Annexin V/PI staining assay (Figure 4C). The percentages of early apoptotic T cells significantly increased with different doses of isovitexin after 24 h of incubation. To define the pathway of apoptosis, western blotting was used to analyze the cleavage of poly (ADP-ribose) polymerase (PARP) and caspase. Strong cleavage of PARP together with activation of caspase-3 and -8 were observed in the Con A-activated T cells treated with isovitexin, which is consistent with the results of animal experiments (Figures 4D,E). These observations indicate that isovitexin inhibits the proliferation of Con A-activated T cells by promoting apoptosis.

### Isovitexin Inhibits the Production of Proinflammatory Cytokines in Con A-Activated T Cells

**FIGURE 5:**
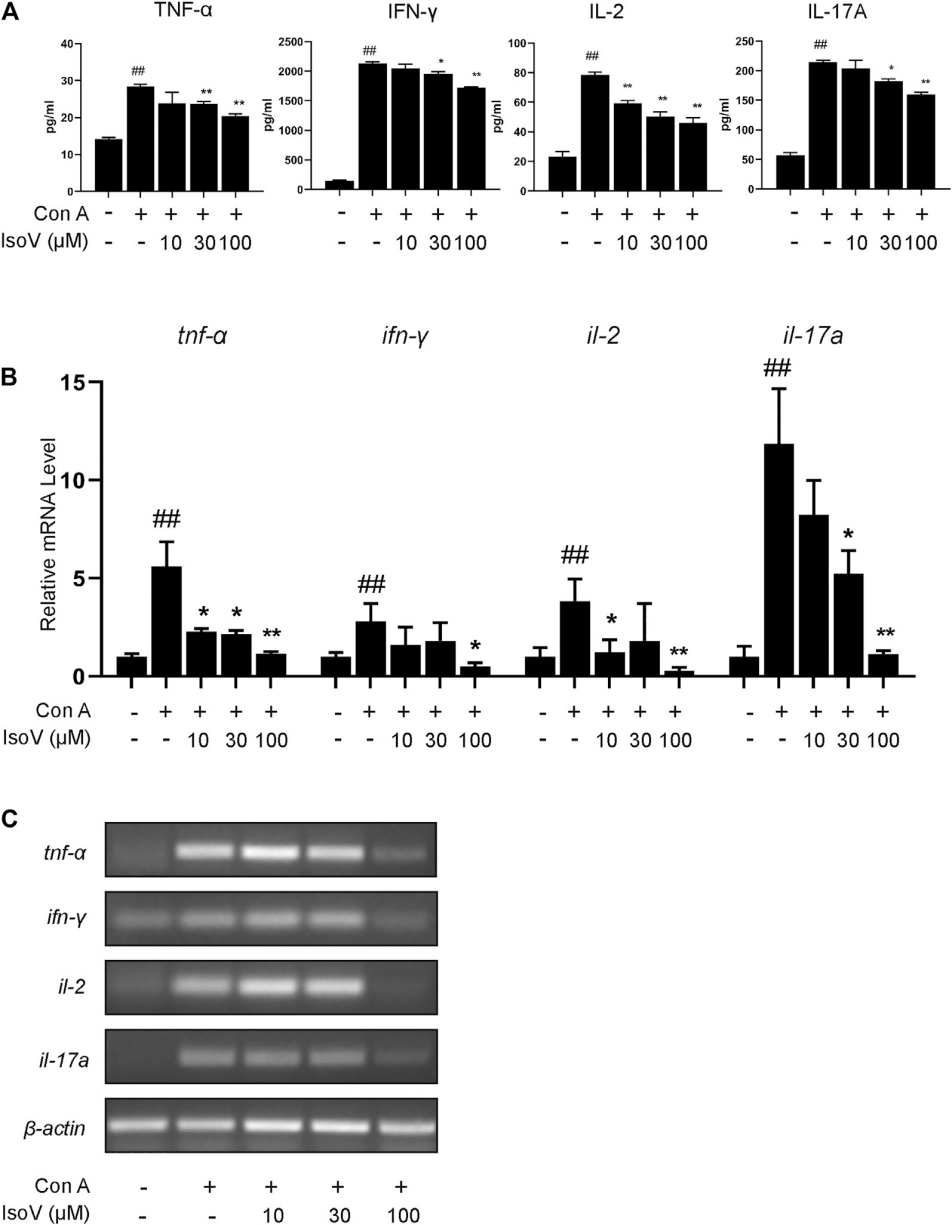
Isovitexin inhibits T cells proinflammatory cytokines production in activated mouse T cells. Con A-activated T cells were treated with different concentrations of isovitexin for 24 h; **(A)** Proinflammatory cytokines, in culture supernatants of the indicated groups, were determined by ELISA; **(B,C)** Relative *tnf-*α*, ifn-*γ*, il-2, il-17a* mRNA level was determined by Q-PCR **(B)** and RT-PCR **(C)**. Data are mean ± SEM and are representative of at least three independent experiments. Significant differences are expressed as ^##^ *p* < 0.01 vs. Blank group, *p < 0.05, **p < 0.01 vs. control (Con A-activated T cells).

Numerous apoptotic cells have been shown to inhibit proinflammatory cytokine production, preventing chronic inflammation (Voll et al., 1997). For this claim, we examined whether isovitexin is linked with the production of proinflammatory cytokines, including TNF-α, IFN-γ, IL-2 and IL-17A*.* ELISA was performed to measure the release of cytokines in the culture supernatant, and RT-PCR was carried out to measure the expression of these cytokines from Con A–activated T cells (Figures 5A–C). Interestingly, at a concentration of 100 μM, isovitexin significantly reduced the levels of proinflammatory cytokines (TNF-α, IFN-γ, IL-2 and IL-17A) at both the mRNA and protein levels.

### The MAPK and STAT Signaling Pathways are Regulated by Isovitexin in Con A-Activated T Cells

**FIGURE 6:**
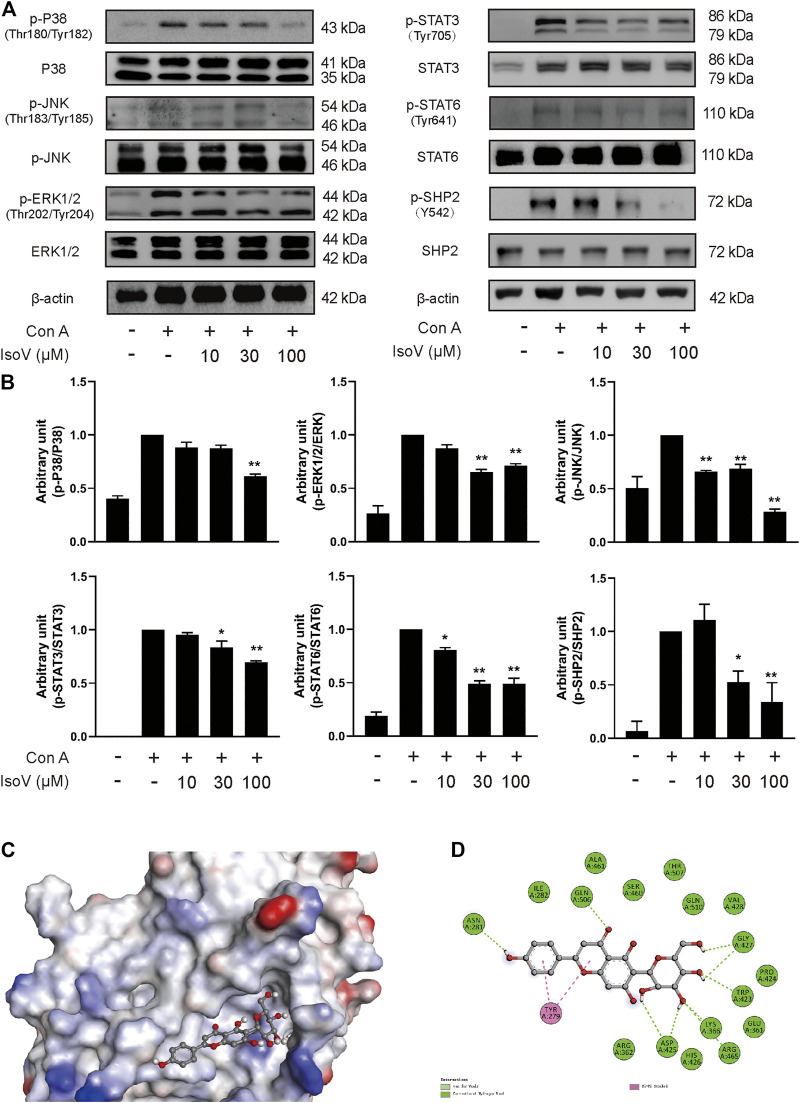
Isovitexin inhibits MAPK, STAT signaling and decreases the phosphorylated SHP2 level in Con A–activated T cells. **(A,B)** T cells isolated from the lymph nodes of Balb/c mice were stimulated with 5 μg/ml Con A and various doses of isovitexin for 24 h. The protein levels of p-P38, p-JNK, p-ERK1/2, p-STAT3, p-STAT6, p-SHP2 in T cells were measured by western blotting, and the relative band density was analyzed using ImageJ Data are mean ± SEM and are representative of at least three independent experiments. The grouping of gels were cropped from different parts of the different gels. Significant differences are expressed as *p < 0.05, **p < 0.01 vs. the Con A group. **(C,D)** The induced fit docking analysis of isovitexin and SHP2 protein (PDB:3o5x). Hydrogen bonds and π-π stacking were indicated with green and pink dot line, respectively.

The MAPK and STAT signaling pathways govern the expression of most proinflammatory genes (Huang et al., 2010; Villarino et al., 2017). To understand how isovitexin modulates inflammatory responses in T cells, we examined the relationship between isovitexin and these pathways. Mechanistically, T cells treated with 100 μM isovitexin exhibited markedly reduced phosphorylation of proteins such as P38, JNK, and ERK1/2 in Con A-induced T cells, which are the central kinases in the MAPK signaling pathway that cause inflammation. On the other hand, isovitexin dose-dependently decreased the level of IκB phosphorylation. The kinase is activated by a highly diverse group of extracellular signals, including inflammatory cytokines, growth factors, and chemokines. Unexpectedly, isovitexin did not affect the expression of p65 in whole cell lysate, and whether p65 is in the core needs to be further proven. Western blotting was also used to explore whether isovitexin decreased the phosphorylation of STAT3, STAT6, and SHP2, which play important roles in the proliferation and differentiation of T cells. Thus, isovitexin serves as a negative regulator of the MAPK and STAT signaling pathways (Figures 6A,B).

### Molecular Docking Analyses of the Interaction Between Isovitexin and SHP2 Protein

Next, we sought to determine the molecular mechanisms by which isovitexin modulates the MAPK and STAT signaling pathways in Con A-activated T cells. We first tested whether isovitexin might interact with SHP2. The interaction between the SHP2 protein and isovitexin was demonstrated by molecular docking analysis. As shown in the figure, there is a certain intensity of interaction between SHP2 and isovitexin, and the best induced-fit docking score is −5.414 kcal/mol, where isovitexin can insert into the active domain of SH2 kinase and interact by hydrogen bonding and π–π interactions (Figure 6C). The docking results suggest that there is obvious π-π conjugation between TYR279, the B ring and the C ring of isovitexin. There may be two hydrogen bond interactions between SHP2 and isovitexin: one formed between the ASN281 residue and 4′-OH on the C ring of isovitexin, and the other formed between GLN and the carbonyl group on the B ring of isovitexin. Indeed, the A ring of isovitexin forms seven kinds of hydrogen bonds with SHP2 with ASP425, LYS366, AGR465, TRP423 and GLY427, which confer stability to the docking conformation (Figure 6D).

### The Effects of Isovitexin on Con A-Activated T Cells can be Reversed by the SHP2-Specific Inhibitor SHP099

**FIGURE 7:**
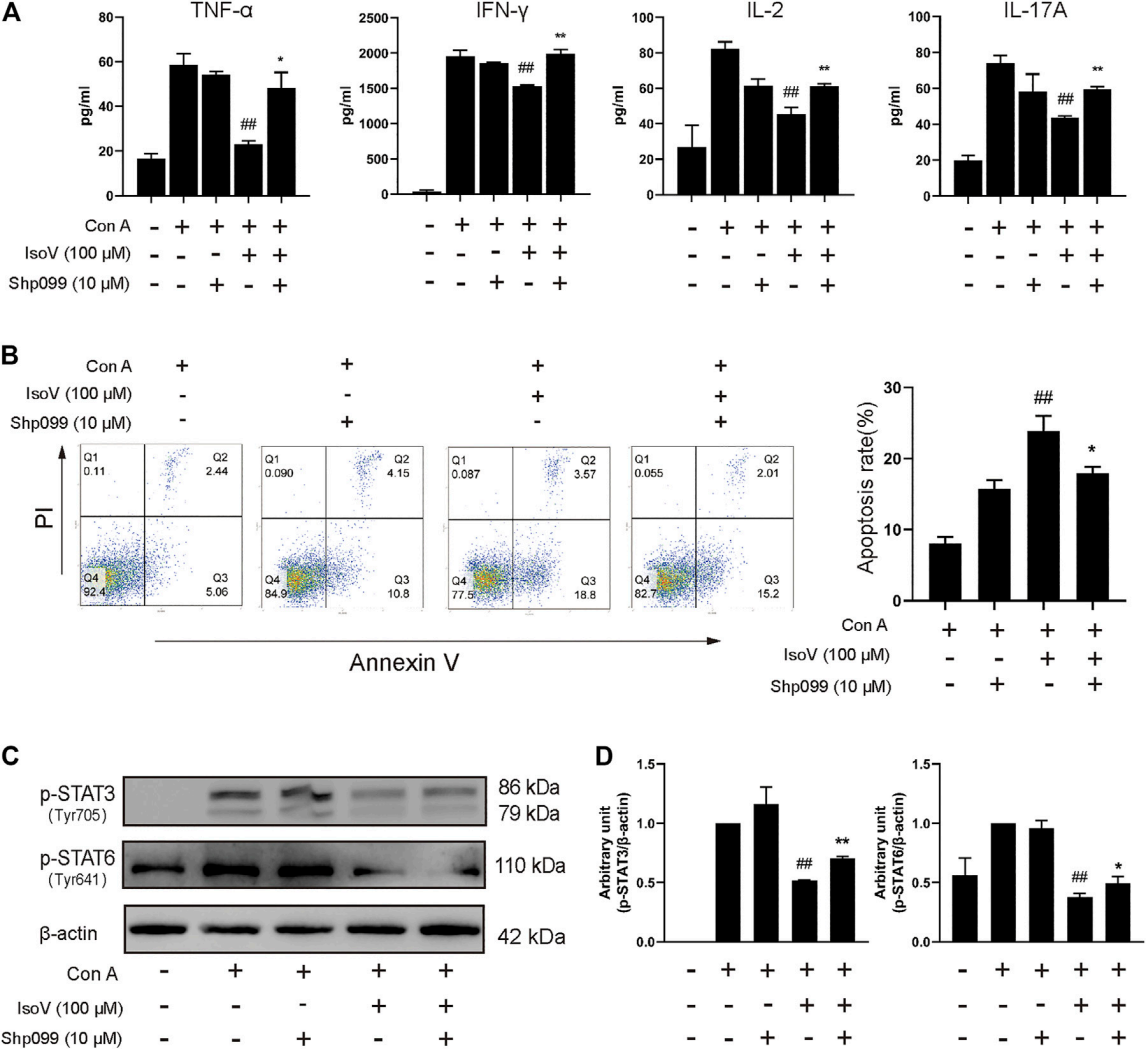
The SHP2-specific inhibitor SHP099 reversed the effects of isovitexin on activated T cells. Con A-activated T cells were treated with 100 μM isovitexin and added with 10 μM SHP2 or not; **(A)** The levels of proinflammatory cytokines in cell culture supernatants were determined using ELISA kits after 24 h cultivation. **(B)** Cells were stained with Annexin V and PI, and the apoptosis of the cells was determined by flow cytometry assay of Annexin V/PI staining. Annexin V positive cells of three independent experiments were shown in column statistics. **(C,D)** Lymph nodes cells were harvested and lyzed and the phosphorylation of STAT3 and STAT6 was evaluated by Western blot. The grouping of blots was cropped from different parts of the same gel. Data are mean ± SEM and are representative of two independent experiments. Significant differences are expressed as ^##^ *p* < 0.01 vs. Con A group, *p < 0.05, **p < 0.01 vs. the isovitexin group.

**FIGURE 8:**
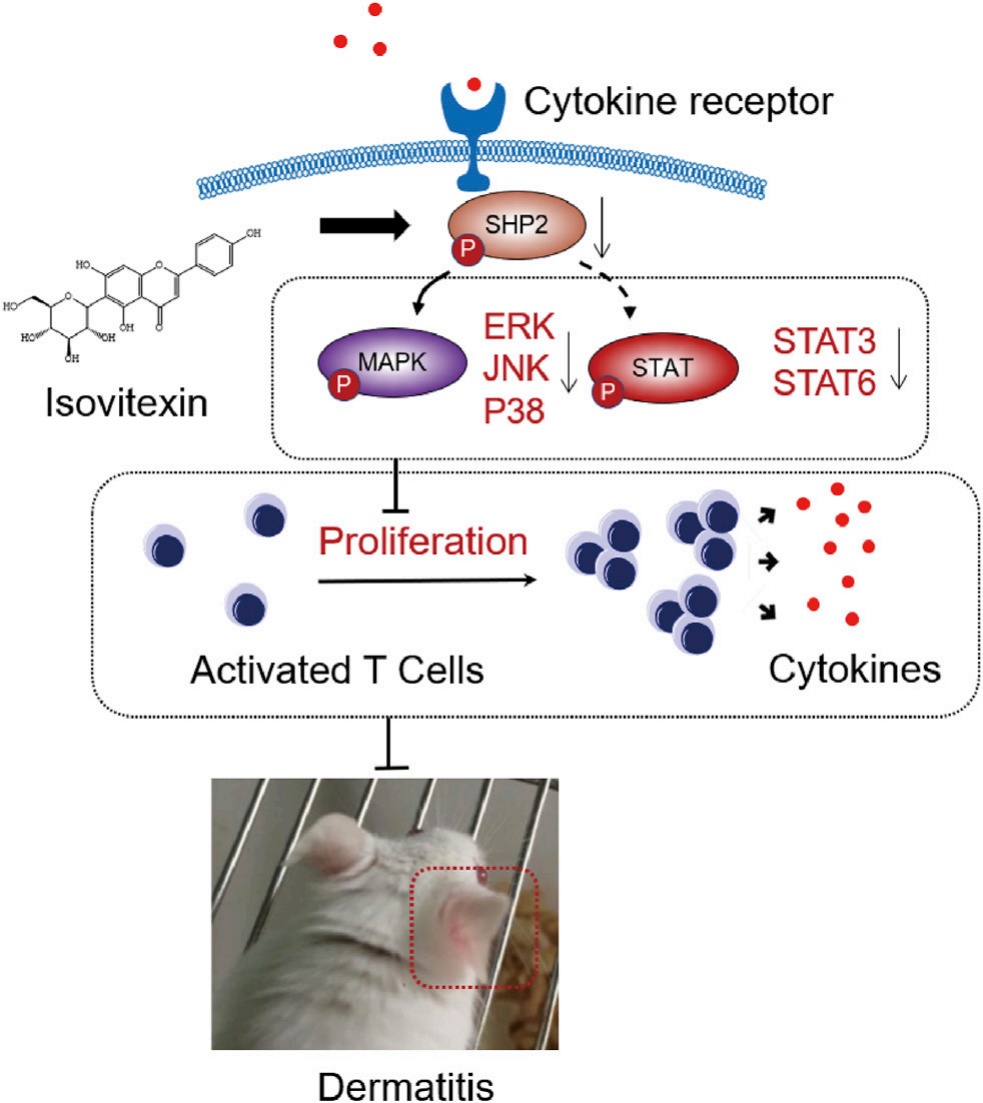
Graphical abstract. When contact hypersensitivity occurs, T cells activated produce proinflammation and chemokine. T cells proliferation and differentiation mediated by receptor tyrosine kinases (RTK) activation, and upregulated its downstream SHP2 protein, MAPK signaling, STAT signaling. On the other hand, isovitexin bind to SHP2 inhibits MAPK signaling, STAT signaling and its dependent deactivating programs in T cells, thus decreasing their production of inflammatory cytokines and reducing the severity of dermatitis.

SHP099, a selective SHP2 inhibitor, binds to protein tyrosine phosphatase domains, inhibiting SHP2 activity through an allosteric mechanism (Pádua et al., 2018). We investigated whether isovitexin suppressed the release of cytokines by regulating SHP2 in Con A-activated T cells, and the effects were examined in SHP099-treated cells. The results showed that the inhibitory effects of isovitexin on T cell proliferation and proinflammatory cytokine production were partially reversed by SHP099 (Figures 7A,B). Moreover, In the SHP099 group, the phosphorylation levels of STAT3 and STAT6 were higher than isovitexin group (Figures 7C,D). The presumed reason is that SHP099 inhibited MAPK signaling pathway, and STAT as a supplementary pathway the phosphorylation level of increased. In summary, our study proved that isovitexin restrains p-SHP2 and controls dermatitis inflammation (Figure 8).

## Discussion

Allergic contact dermatitis is a skin disease caused by environmental or occupational allergens. As a general concept of the antigen presentation process in the sensitization phase of ACD, allergens activate innate immunity through keratinocyte release of proinflammatory cytokines and chemokines to recruit T cells. Hapten-specific T cells are guided to inflammatory sites and produce cytokines, such as TNF-α, IFN-γ and IL-17A. In turn, these cytokines then stimulate skin-resident cells, which lead to further recruitment of T cells and induce an inflammatory cascade (Honda et al., 2013; Leonard and Guttman-Yassky, 2019). Indeed, dysregulation of the skin immune system occurs in nearly all ACD cases, which highlights the importance of appropriate immune regulation in preventing ACD. However, studies of contact-hypersensitivity mouse models have mostly used synthetic experimental allergens, such as DNFB. Different allergens cause widely divergent immune responses, resulting in the eventual failure of the therapeutic strategies for ACD. We therefore optimized a mouse model of GA-induced ACD to identify inflammation and ear swelling. We identified a critical role of proinflammatory cytokines such as TNF-α, IFN-γ, IL-2 and IL-17A in skin inflammation in this model (Figures 3A,B) and revealed that these cytokines may be regulated by SHP2 *in vitro* (Figure 6A).

As an environmental allergen, GA-induced ACD is usually treated with NSAIDs or immunosuppressive drugs, and long-term use may result in serious side effects, including effects on the gastrointestinal tract and infection susceptibility (Rosa et al., 2016). In this context, new pharmacological strategies are being sought, such as resolution of inflammation with fewer side effects. In the present study, we have provided proof that CLE, especially isovitexin, may dampen proinflammatory signaling and the clearance of proinflammatory mediators to attenuate inflammation. In conclusion, isovitexin acts as an inflammatory inhibitor to ameliorate GA-induced ACD (Figures 1, 2).

Isovitexin is the most abundant flavone in the leaf of *Celtis sinensis*. It has anti-inflammatory pharmacological properties. In a previous study, isovitexin attenuated the LPS-induced phosphorylation of all three MAPKs, reduced NF-κB activation and promoted M2 polarization in macrophages (Lv et al., 2016; Liu et al., 2019). However, few studies targeting the anti-inflammatory effects of T lymphocytes by isovitexin treatment have been reported. To reveal the regulatory effects of isovitexin on ACD, which is characterized by the Th1 response (Owen et al., 2018), we used T cells from mouse lymph node cells under Con A stimulation. In this study, we found that isovitexin dose-dependently upregulated apoptosis and suppressed the cytokines TNF-α, IFN-γ, IL-2 and IL-17A (Figure 5). This result is consistent with animal experiments, indicating that isovitexin exerts an immunomodulatory effect when facing an inflammatory challenge.

To further reveal the underlying mechanisms of isovitexin on T cell apoptosis and signaling pathways. We further examined the effects of isovitexin on cleaved caspase-3, cleaved caspase-8, MAPK signaling and STAT signaling in activated T cells. In this study, we have provided several lines of evidence that suggest that cleaved caspase-3 and cleaved caspase-8 are significantly enhanced by isovitexin treatment in a dose-dependent manner (Figure 4). These cleaved proteins lead to increasing apoptosis. MAPK signaling and STAT signaling, along with countless studies linking to inflammatory pathologies, provide the rationale for applying potent inhibitors in the treatment of immune-system-mediated diseases (Villarino et al., 2017; Yahfoufi et al., 2018). Our research result is somewhat consistent with a previous report in which isovitexin normalized the phosphorylation of all three MAPKs and the STAT signaling pathway. In addition, isovitexin dampens proinflammatory signaling and promotes the resolution of inflammation (Figure 6).

SHP2, a nonreceptor protein tyrosine phosphatase, was the first reported oncogenic tyrosine phosphatase and has attracted much attention. The function of SHP2 was reported to regulate cell survival and proliferation primarily through activation of the RAS-ERK signaling pathway and to mediate the immune checkpoint pathways (Chen et al., 2016). Moreover, SHP2 is an important mediator in lupus erythematosus, ERK/MAPK signaling normalization and reduced production of IFN-γ and IL-17a cytokines involved in inflammation can occur by directly inhibiting SHP2 (Wang et al., 2016). Meanwhile, SHP2 is required for full activation of the JAK/STAT pathway, a major signaling cascade in inflammation (Frankson et al., 2017). SHP2 has been characterized as a positive regulator of JAK2/STAT3 signaling in rheumatoid arthritis diseases (Ganesan and Rasool, 2017). Here, molecular docking showed that isovitexin may bind to the PTP domain with hydrogen bonding and π–π interactions (Figure 6B). Western blotting experiments suggested that isovitexin normalized Src-homology two domain-containing phosphatase tyrosine (Y542) phosphatase, which upregulates phosphatase activity (Jiang et al., 2019). Indeed, downregulated SHP2 reduces the downstream MAPK and STAT signaling pathways, which decreases the level of proinflammatory cytokine release and ameliorates ACD. It also prevents the T cell-decreased MAPK signaling, which would cause autoreactivity and autoimmunity (Gorelik et al., 2007). In summary, isovitexin blockade of SHP2 may be a novel and effective therapy for the treatment of patients with GA-induced ACD.

SHP099, an inhibitor that binds to the allosteric site of SHP2 and stabilizes the closed form of SHP2 by interacting with the N-SH2 and PTP domains, has been used to provide several lines of evidence, which suggested functional expression and a physiological signaling role of SHP2 in Con A-active T cells. First, the Annexin V/PI staining results showed that T cells treated with isovitexin and SHP099 had an increased proportion of living cells vs. the isovitexin group alone (Figure 7B). These results indicated that isovitexin promotes T lymphocyte apoptosis by downregulating SHP2 activation. Moreover, SHP099 also reversed the inhibitory effects of isovitexin on MAPK and STAT signaling (Figure 7C). Our results indicate that isovitexin exerts SHP2-dependent inhibitory effects in Con A-activated T cells.

In summary, our study demonstrates that isovitexin from Celtis sinensis is a potential therapeutic agent against GA-induced allergic contact dermatitis. The MAPK and STAT signaling pathways can be regulated by isovitexin, and SHP2 may be a potential anti-inflammatory target of isovitexin in T cells. Isovitexin can be used to solve anti-inflammatory problems induced by GA.

## Data Availability

The original contributions presented in the study are included in the article/Supplementary Material, further inquiries can be directed to the corresponding author.
