# Supplementary figures and images for: Isovitexin Inhibits Ginkgolic Acids-Induced Inflammation Through Downregulating SHP2 Activation
Source: Front Pharmacol. 2021 Aug 11;12:630320. doi: 10.3389/fphar.2021.630320 (PMC8385789; doi:10.3389/fphar.2021.630320)

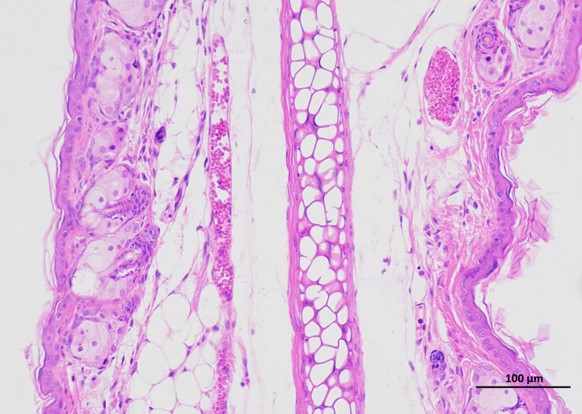

Supplement: Supplementary file 1 [file DataSheet1.ZIP › raw date/raw date/figure1/H&E/1.jpg]

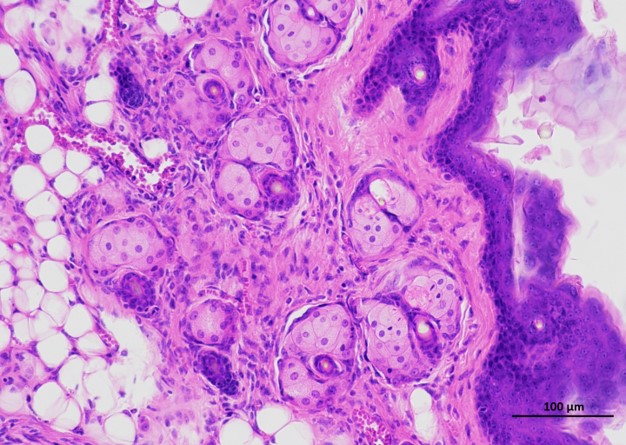

Supplement: Supplementary file 1 [file DataSheet1.ZIP › raw date/raw date/figure1/H&E/2.jpg]

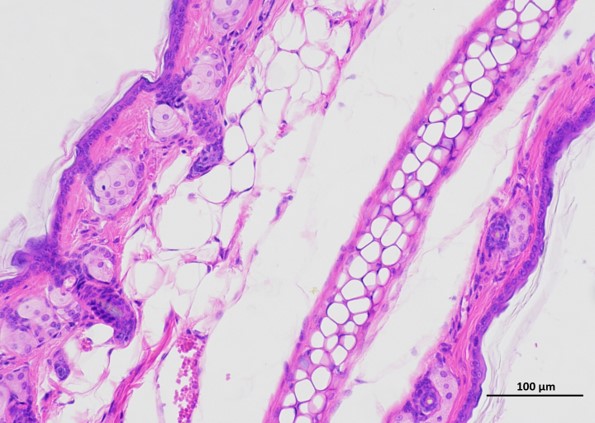

Supplement: Supplementary file 1 [file DataSheet1.ZIP › raw date/raw date/figure1/H&E/3.jpg]

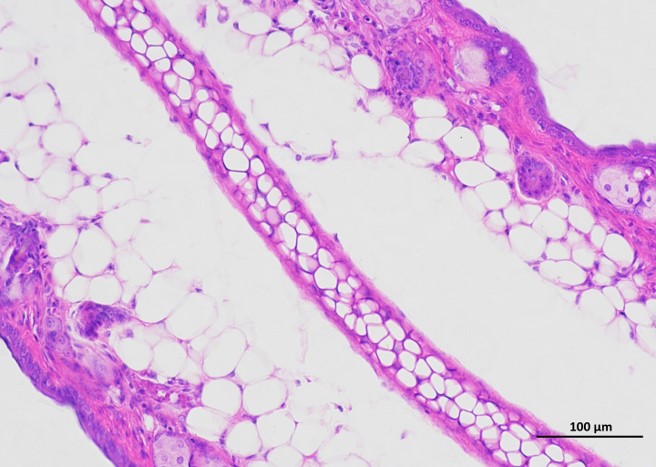

Supplement: Supplementary file 1 [file DataSheet1.ZIP › raw date/raw date/figure1/H&E/4.jpg]

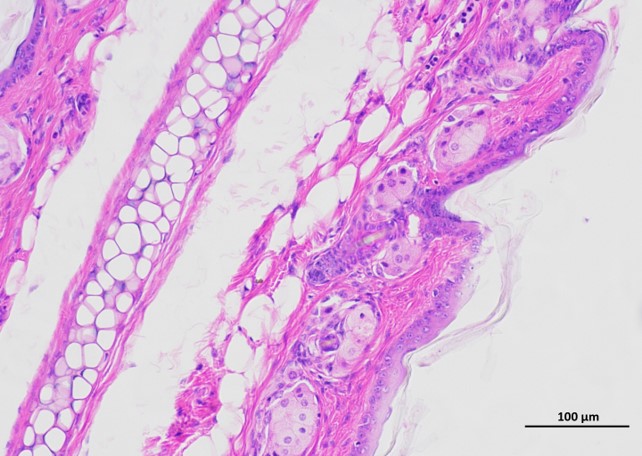

Supplement: Supplementary file 1 [file DataSheet1.ZIP › raw date/raw date/figure1/H&E/5.jpg]

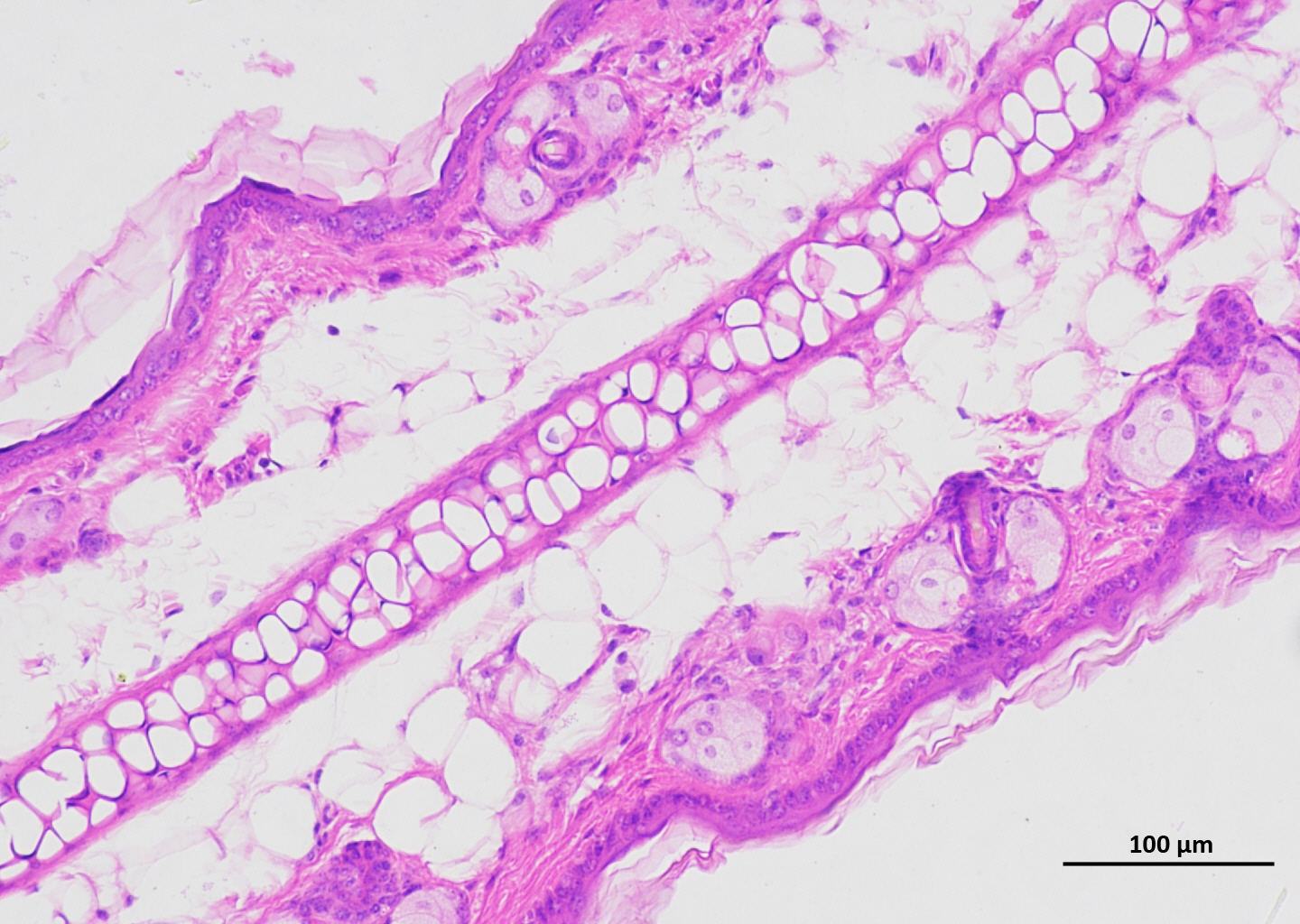

Supplement: Supplementary file 1 [file DataSheet1.ZIP › raw date/raw date/figure2/H&E/1.jpg]

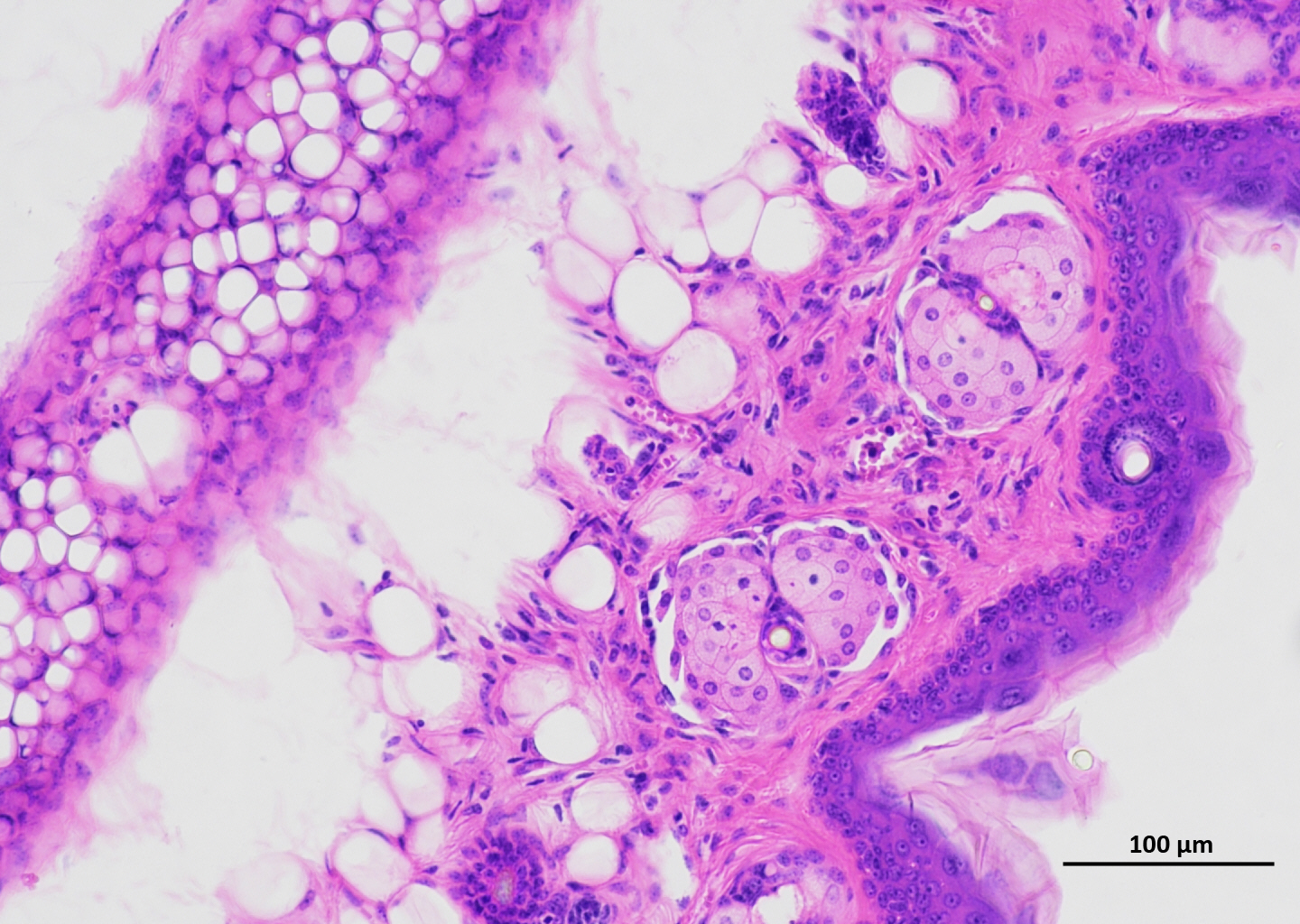

Supplement: Supplementary file 1 [file DataSheet1.ZIP › raw date/raw date/figure2/H&E/2.jpg]

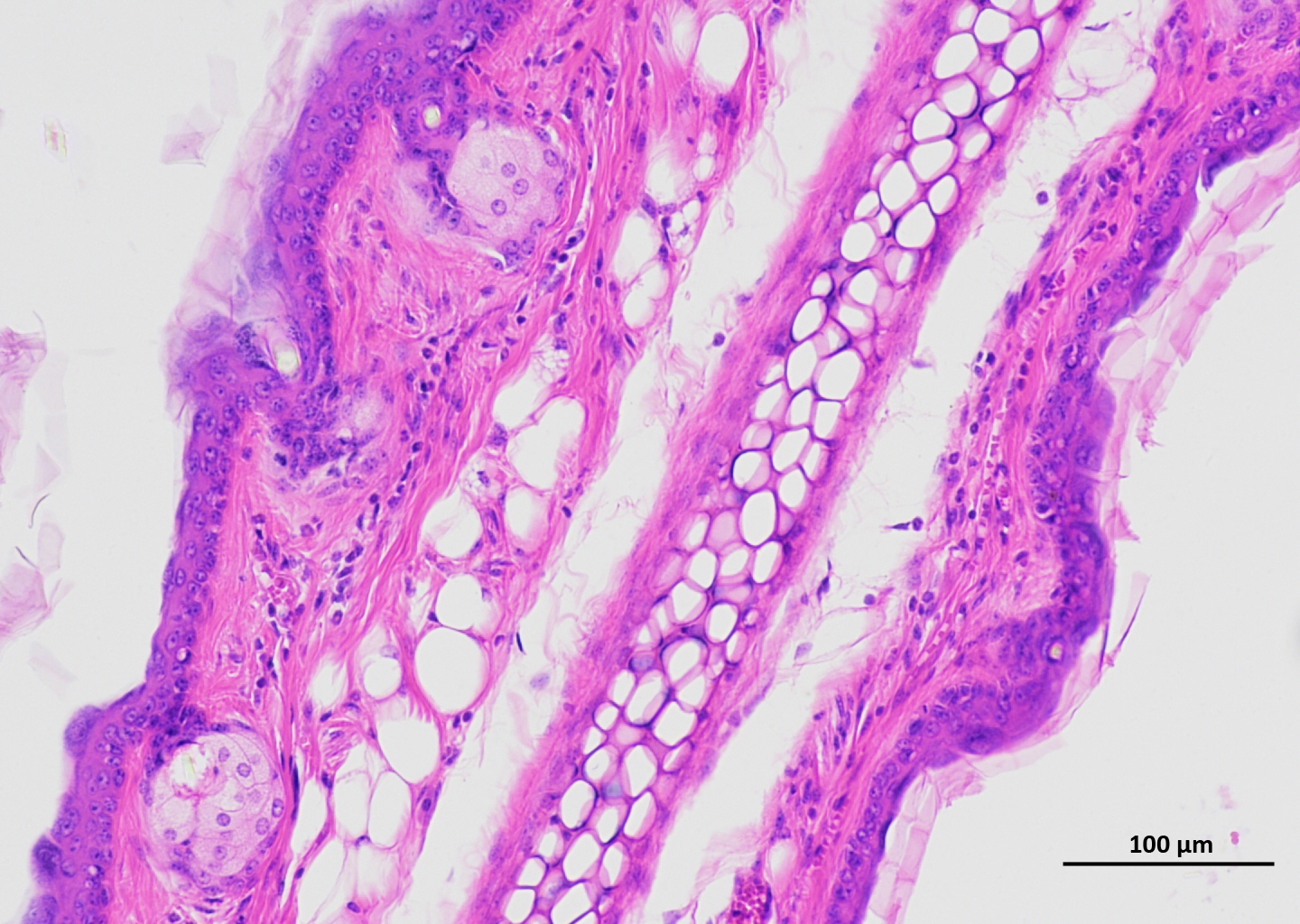

Supplement: Supplementary file 1 [file DataSheet1.ZIP › raw date/raw date/figure2/H&E/3.jpg]

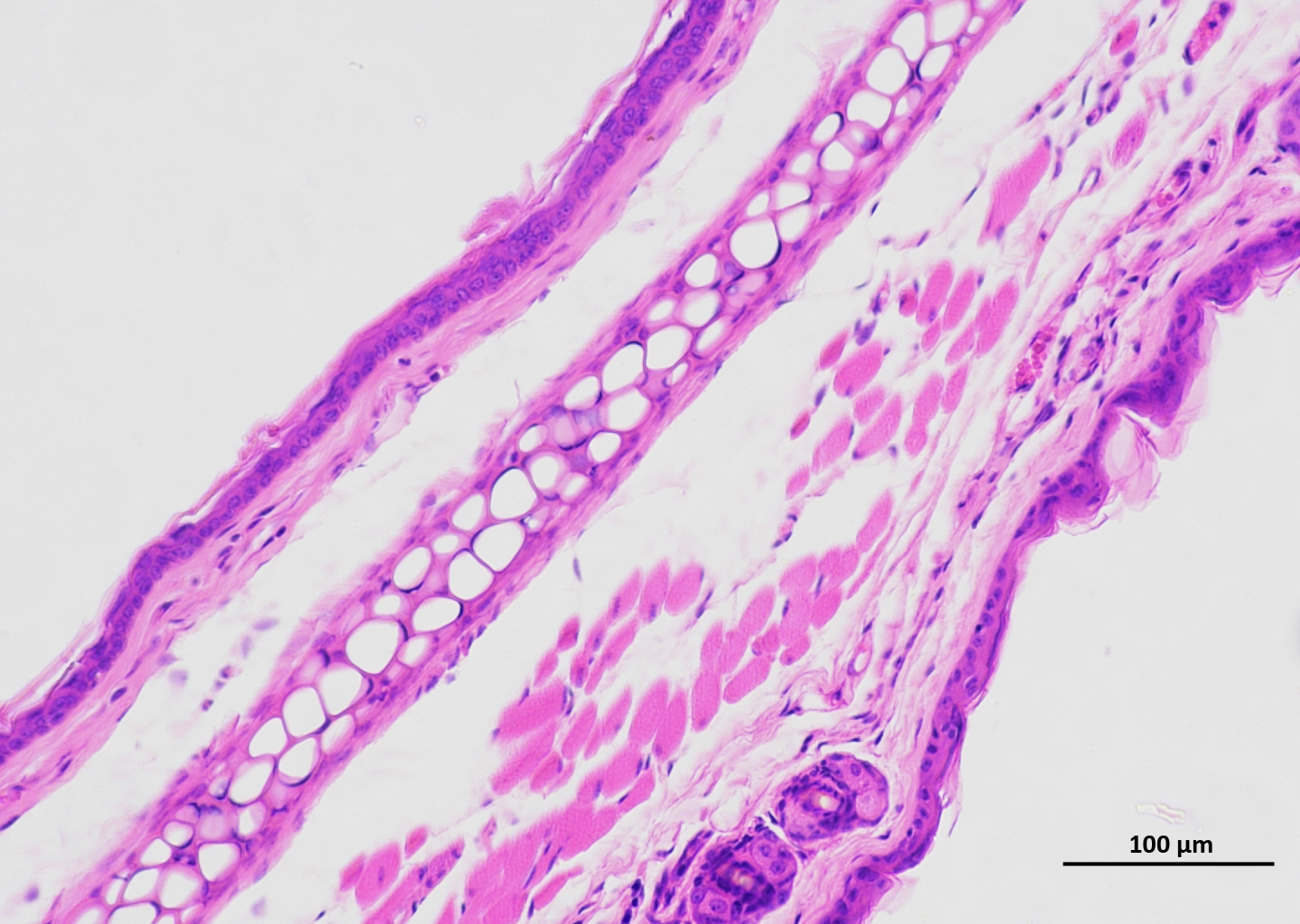

Supplement: Supplementary file 1 [file DataSheet1.ZIP › raw date/raw date/figure2/H&E/4.jpg]

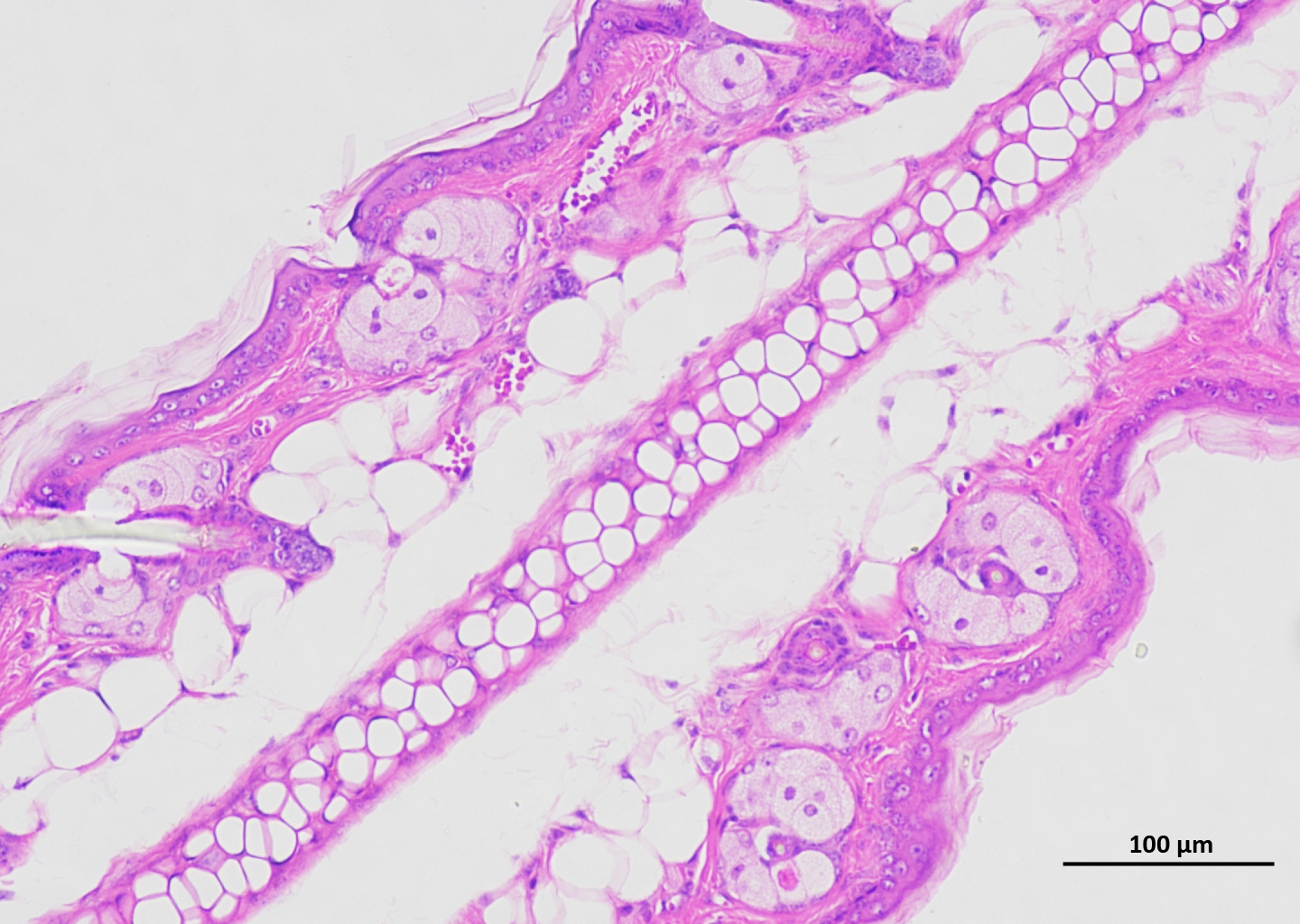

Supplement: Supplementary file 1 [file DataSheet1.ZIP › raw date/raw date/figure2/H&E/5.jpg]

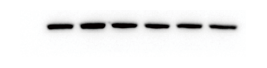

Supplement: Supplementary file 1 [file DataSheet1.ZIP › raw date/raw date/figure3/Figure 3D/akt.png]

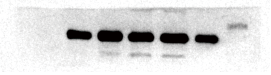

Supplement: Supplementary file 1 [file DataSheet1.ZIP › raw date/raw date/figure3/Figure 3D/b-actin.png]

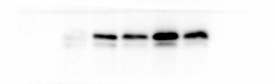

Supplement: Supplementary file 1 [file DataSheet1.ZIP › raw date/raw date/figure3/Figure 3D/c-caspase3.png]

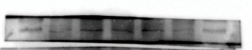

Supplement: Supplementary file 1 [file DataSheet1.ZIP › raw date/raw date/figure3/Figure 3D/c-parp.png]

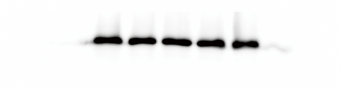

Supplement: Supplementary file 1 [file DataSheet1.ZIP › raw date/raw date/figure3/Figure 3D/caspase3.png]

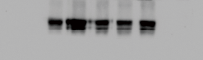

Supplement: Supplementary file 1 [file DataSheet1.ZIP › raw date/raw date/figure3/Figure 3D/erk.png]

Figure 3D


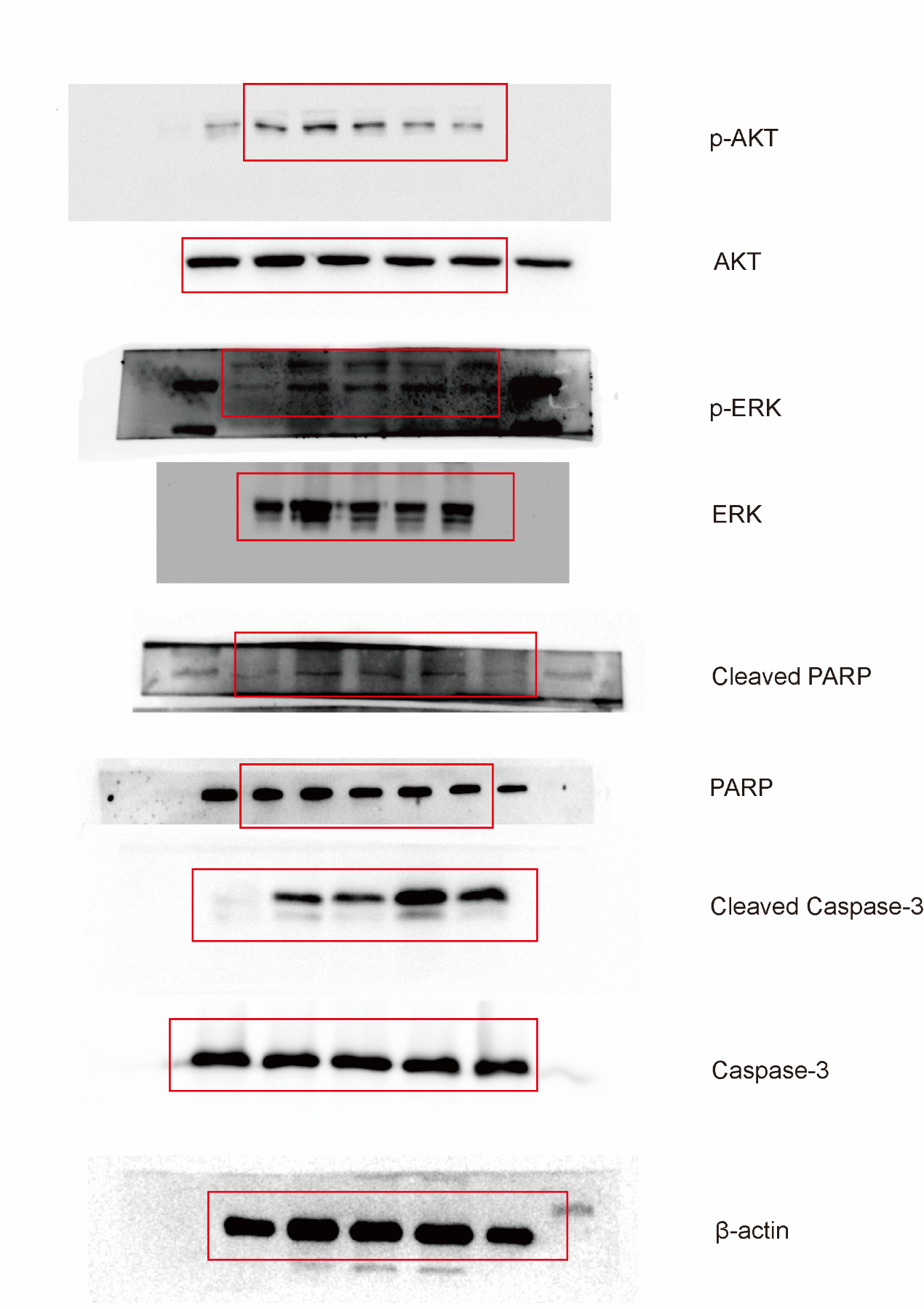

Supplement: Supplementary file 1 [file DataSheet1.ZIP › raw date/raw date/figure3/Figure 3D/Figure 3D.docx]

Figure 3D

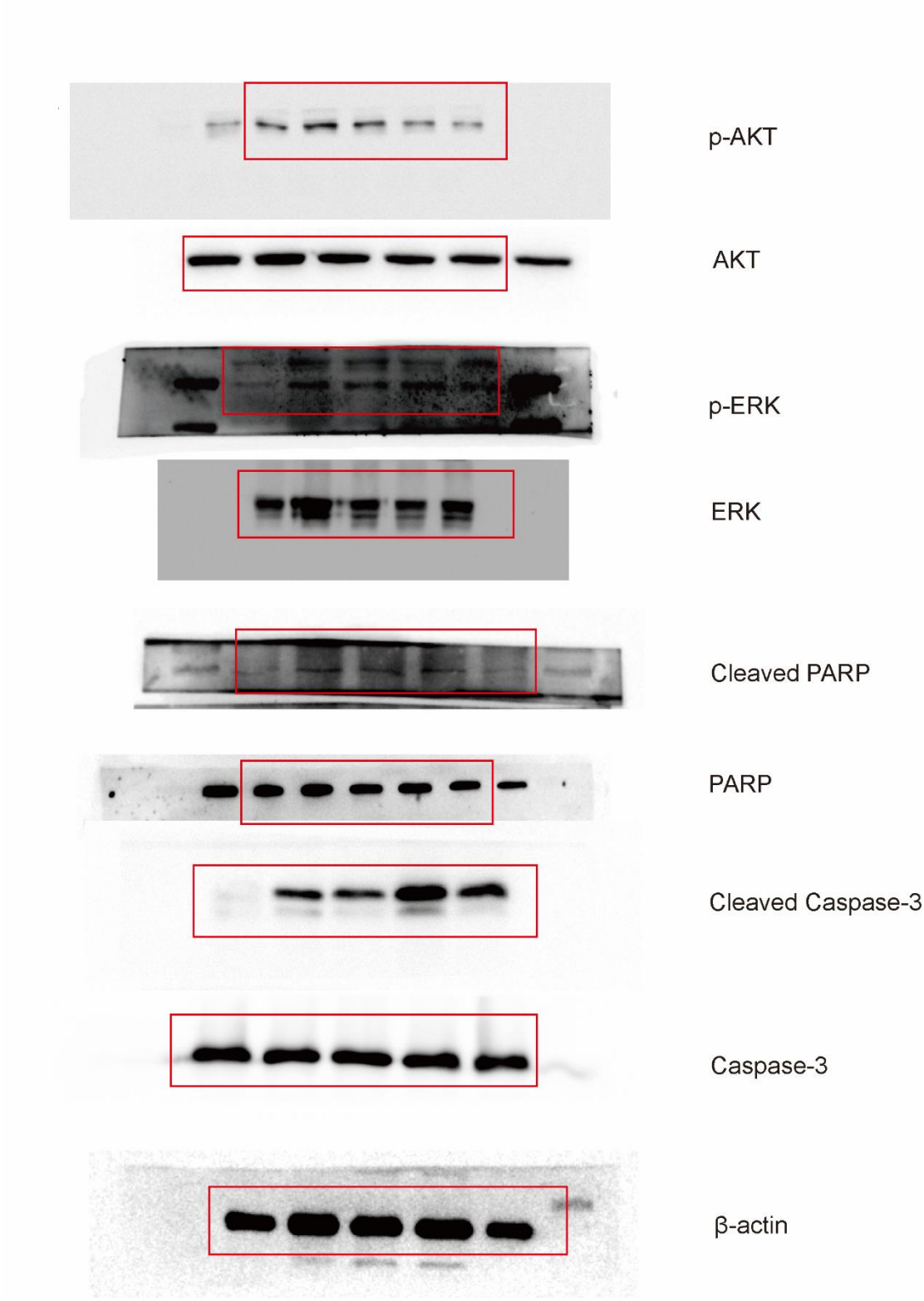

Supplement: Supplementary file 1 [file DataSheet1.ZIP › raw date/raw date/figure3/Figure 3D/Figure 3D.pdf]

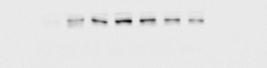

Supplement: Supplementary file 1 [file DataSheet1.ZIP › raw date/raw date/figure3/Figure 3D/p-akt.jpg]

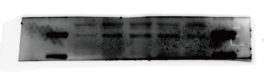

Supplement: Supplementary file 1 [file DataSheet1.ZIP › raw date/raw date/figure3/Figure 3D/p-erk.png]

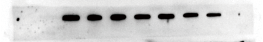

Supplement: Supplementary file 1 [file DataSheet1.ZIP › raw date/raw date/figure3/Figure 3D/parp.png]

Figure 3B


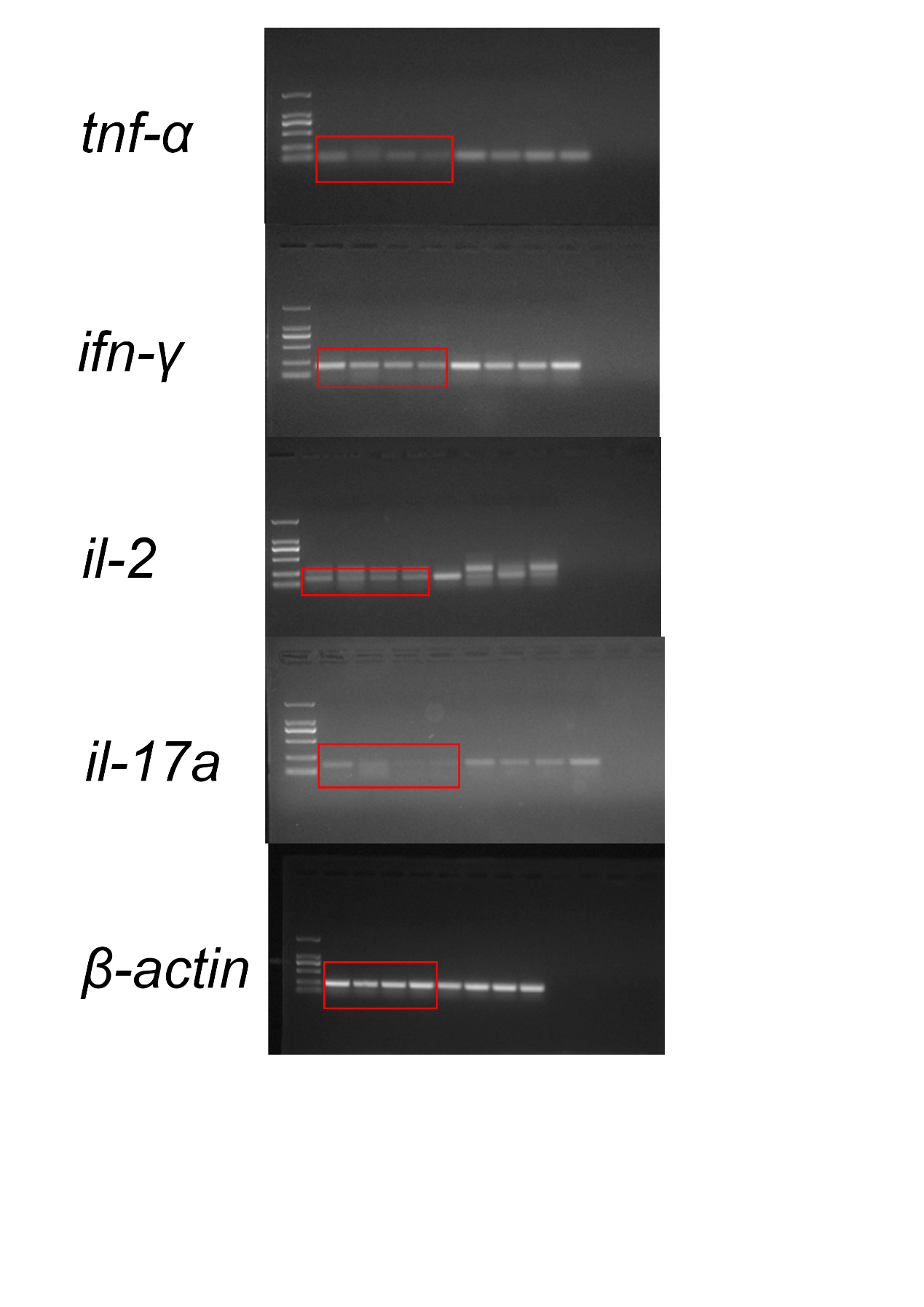

Supplement: Supplementary file 1 [file DataSheet1.ZIP › raw date/raw date/figure3/RT-PCR.docx]

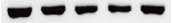

Supplement: Supplementary file 1 [file DataSheet1.ZIP › raw date/raw date/figure4/Figure 4D/akt.png]

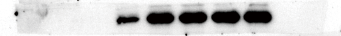

Supplement: Supplementary file 1 [file DataSheet1.ZIP › raw date/raw date/figure4/Figure 4D/b-actin.png]

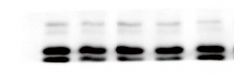

Supplement: Supplementary file 1 [file DataSheet1.ZIP › raw date/raw date/figure4/Figure 4D/c-caspase3.png]

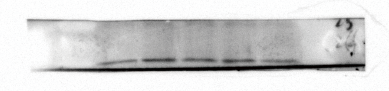

Supplement: Supplementary file 1 [file DataSheet1.ZIP › raw date/raw date/figure4/Figure 4D/c-caspase8.png]

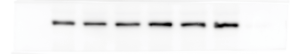

Supplement: Supplementary file 1 [file DataSheet1.ZIP › raw date/raw date/figure4/Figure 4D/c-parp.png]

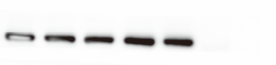

Supplement: Supplementary file 1 [file DataSheet1.ZIP › raw date/raw date/figure4/Figure 4D/caspasr3.png]

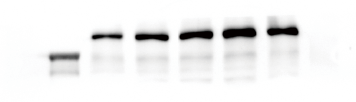

Supplement: Supplementary file 1 [file DataSheet1.ZIP › raw date/raw date/figure4/Figure 4D/caspasr8.png]

Figure 4D


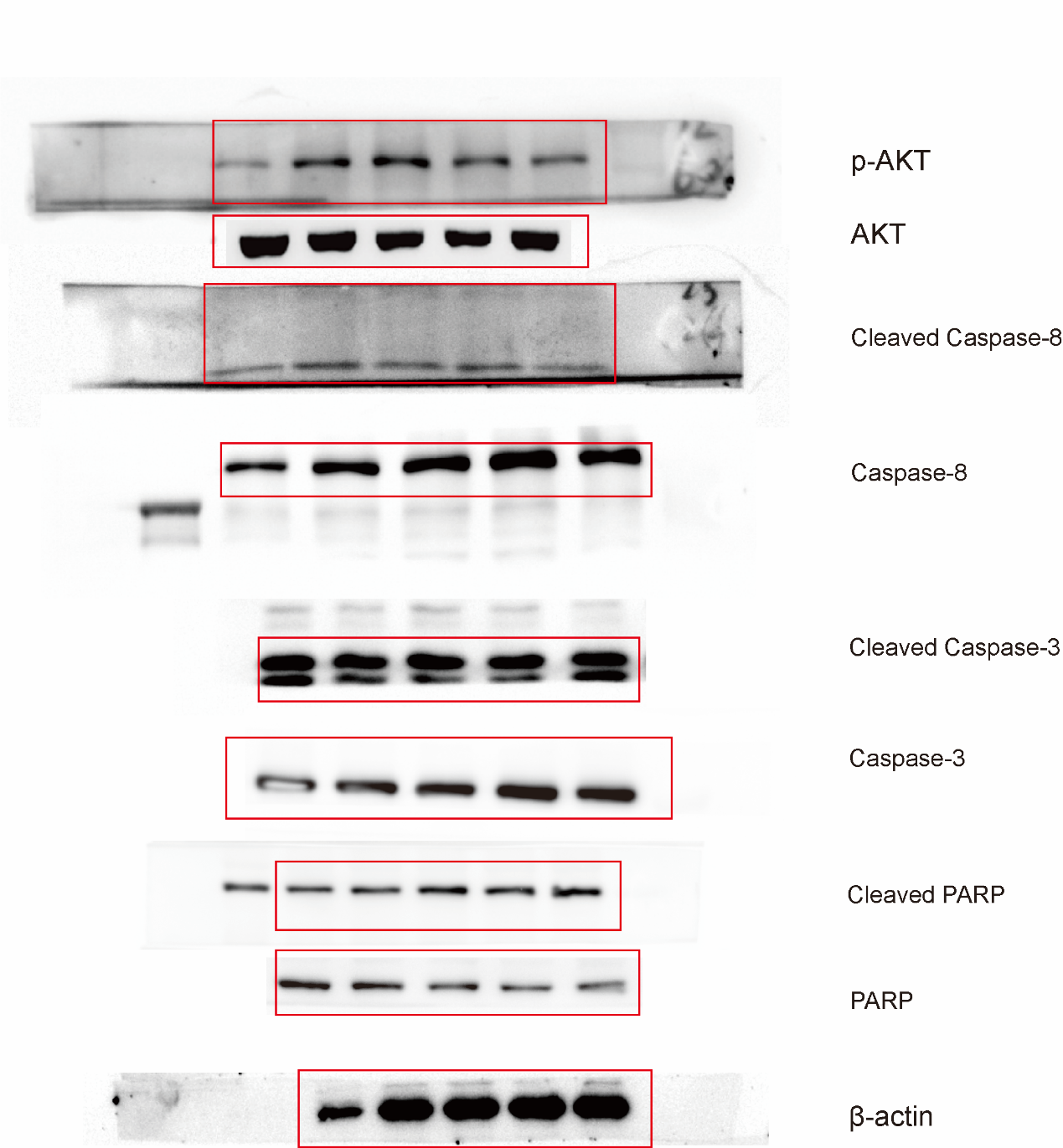

Supplement: Supplementary file 1 [file DataSheet1.ZIP › raw date/raw date/figure4/Figure 4D/figure 4D.docx]

Figure 4D

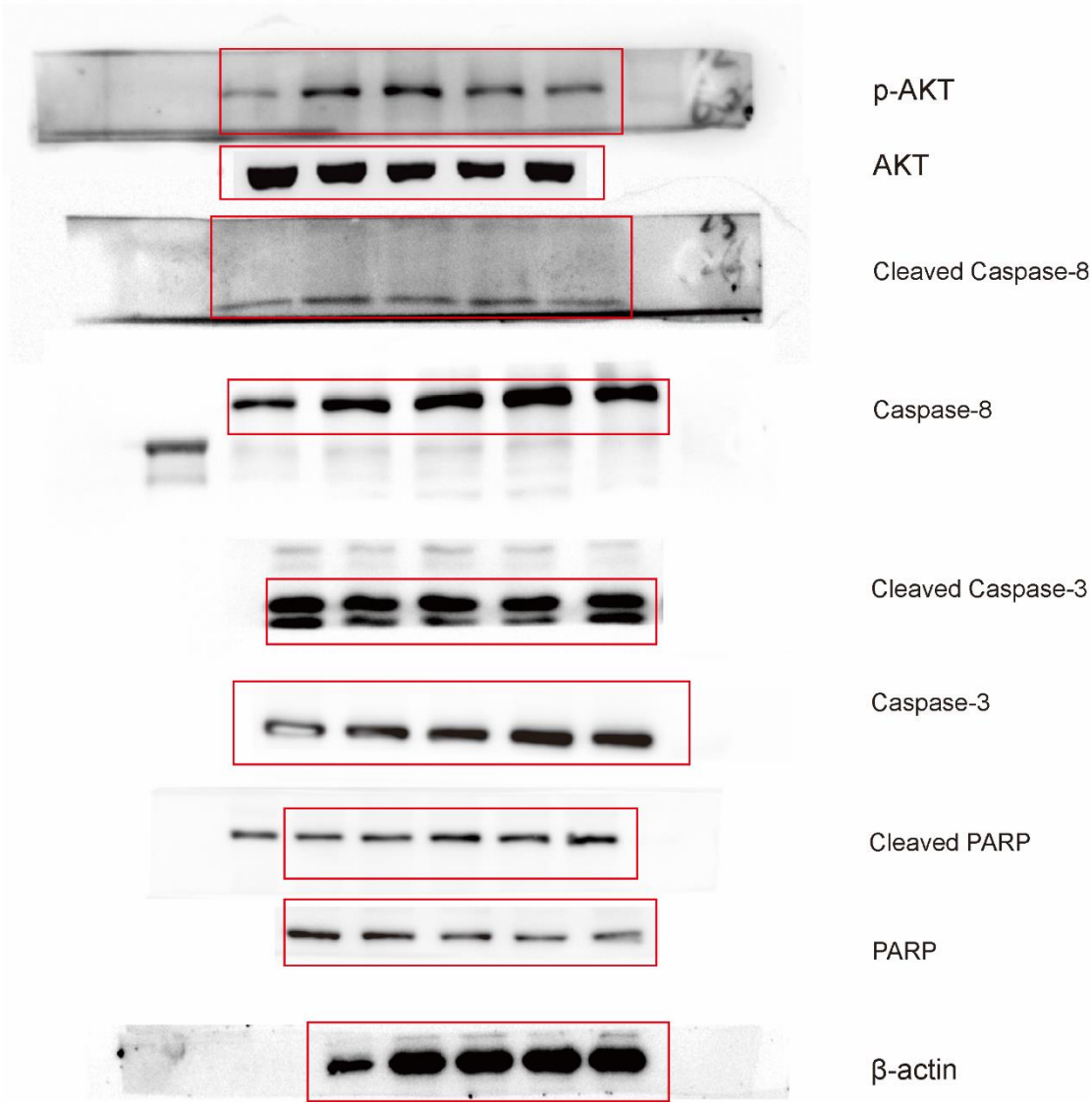

Supplement: Supplementary file 1 [file DataSheet1.ZIP › raw date/raw date/figure4/Figure 4D/figure 4D.pdf]

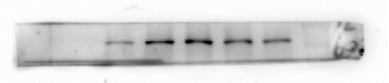

Supplement: Supplementary file 1 [file DataSheet1.ZIP › raw date/raw date/figure4/Figure 4D/p-akt.png]

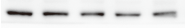

Supplement: Supplementary file 1 [file DataSheet1.ZIP › raw date/raw date/figure4/Figure 4D/parp.png]

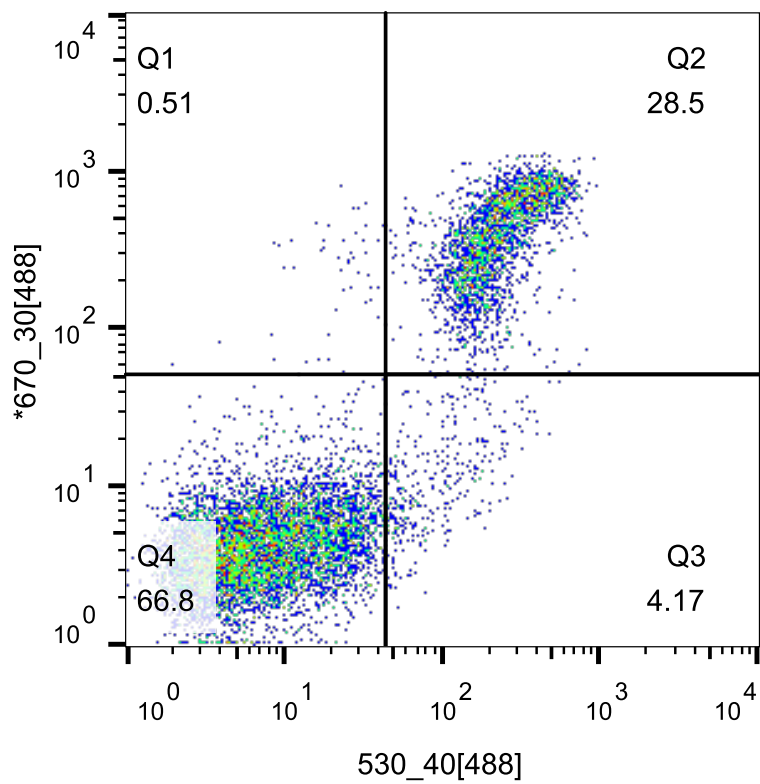

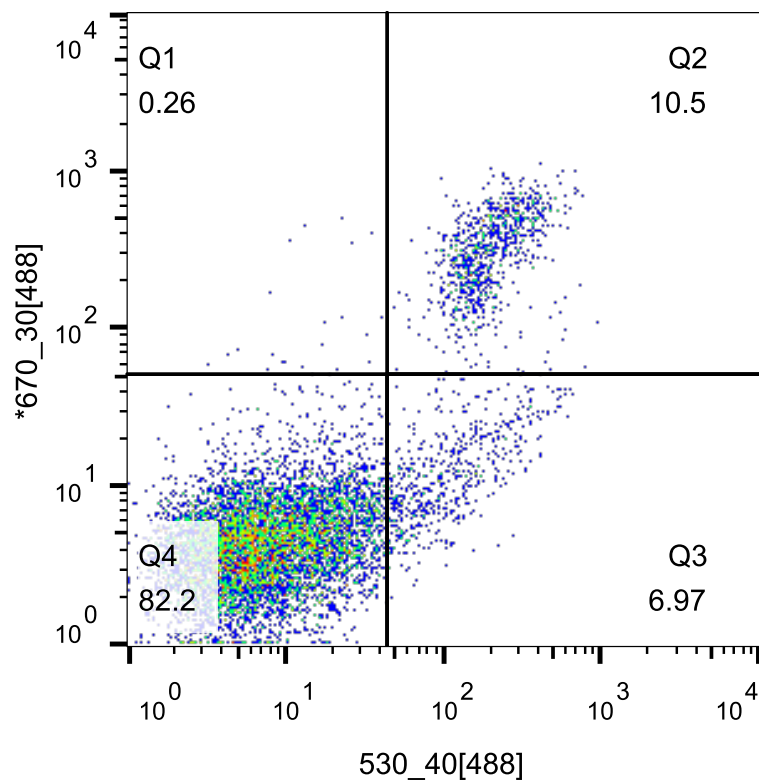

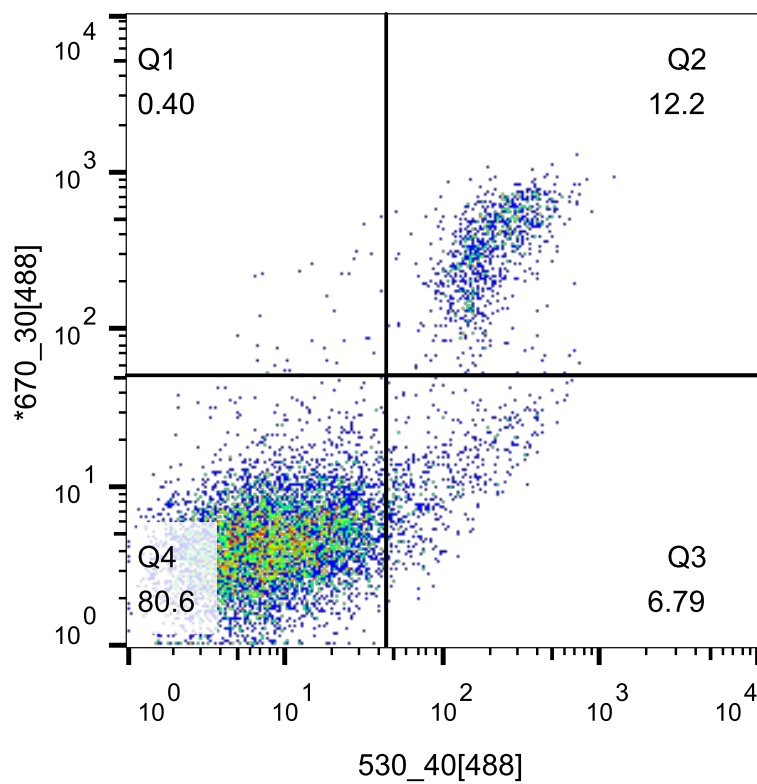

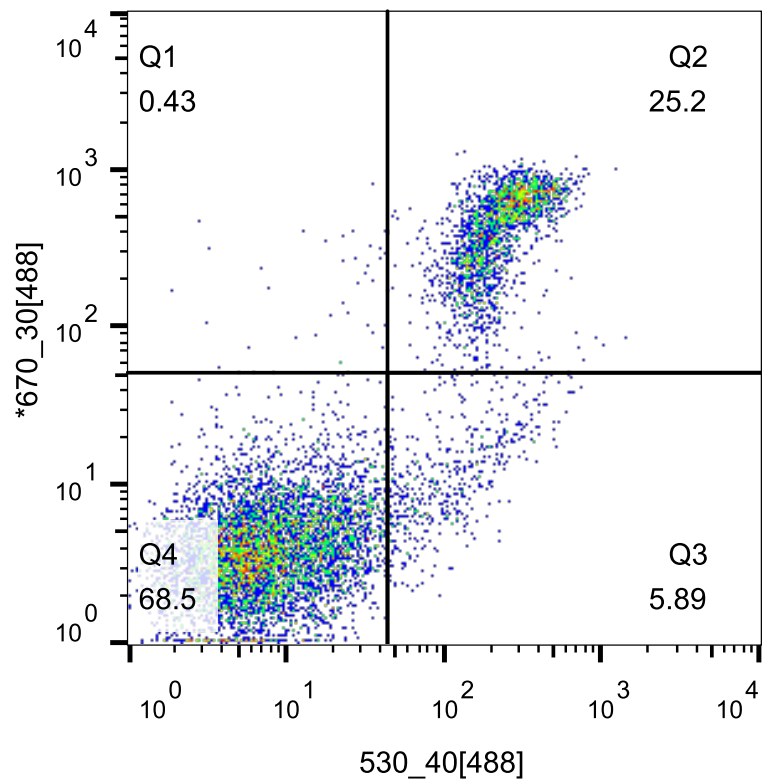

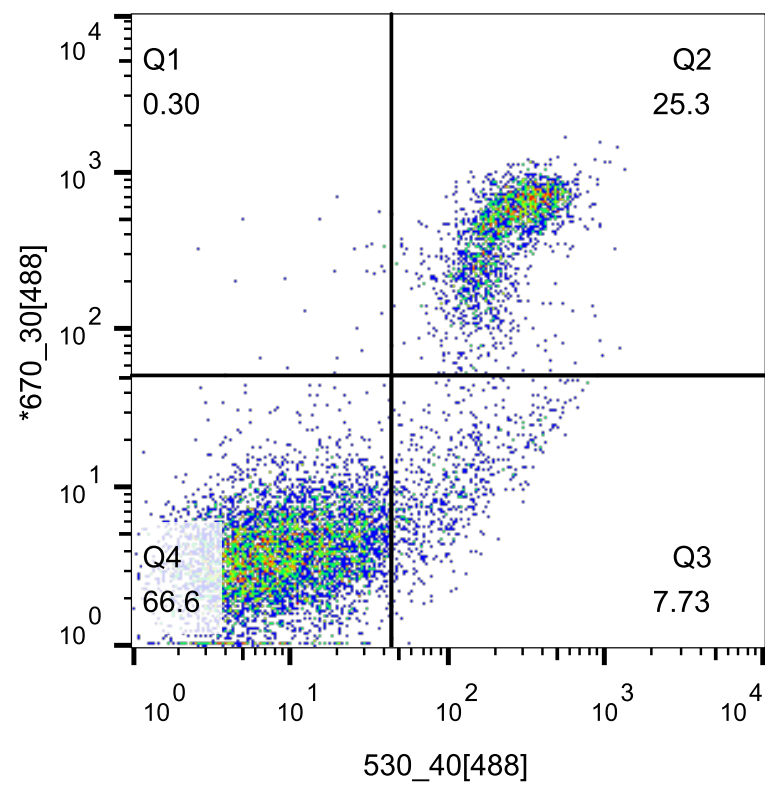

Supplement: Supplementary file 1 [file DataSheet1.ZIP › raw date/raw date/figure4/flow/20210617-Layout.pdf]

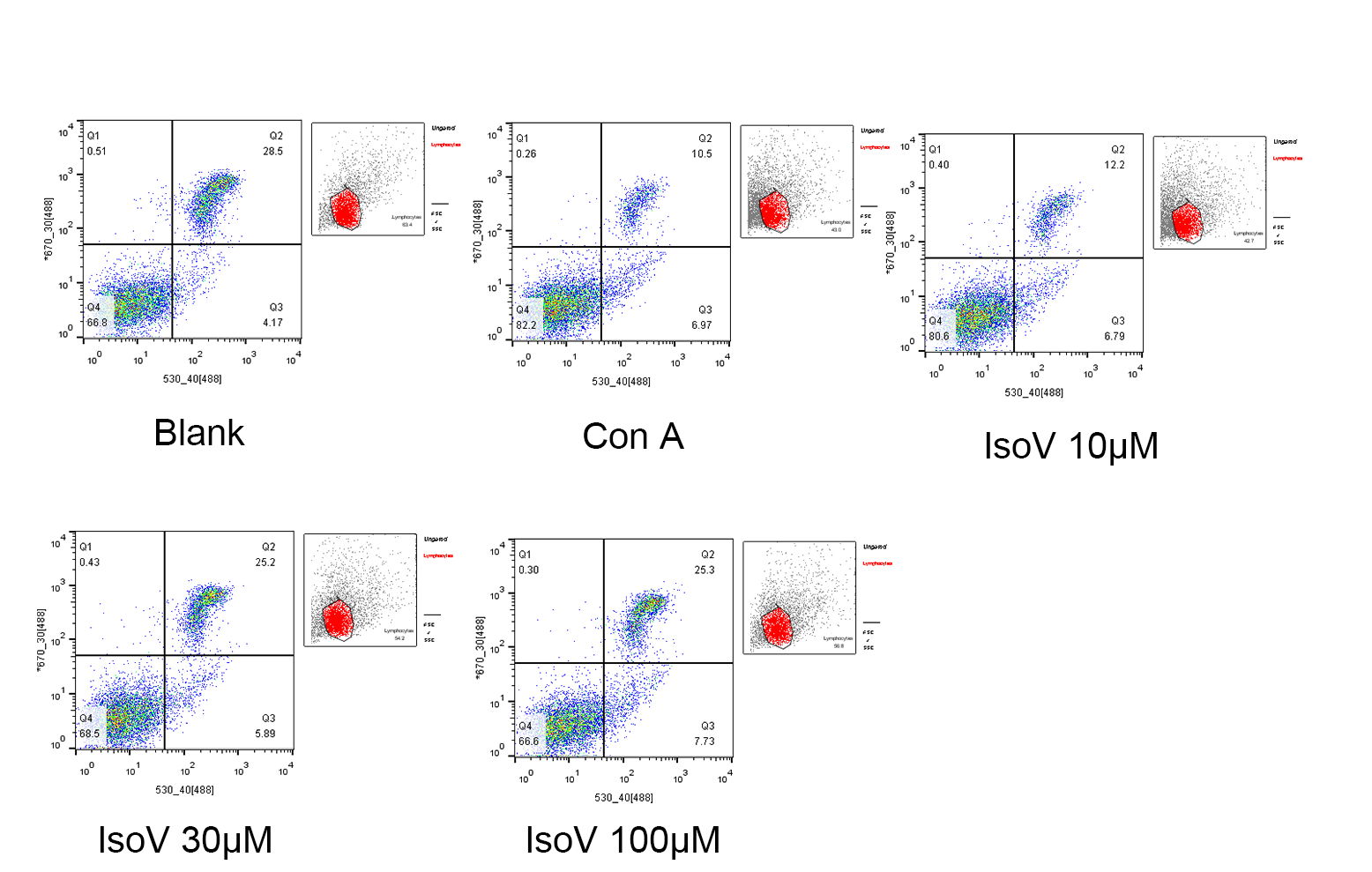

Supplement: Supplementary file 1 [file DataSheet1.ZIP › raw date/raw date/figure4/flow.png]

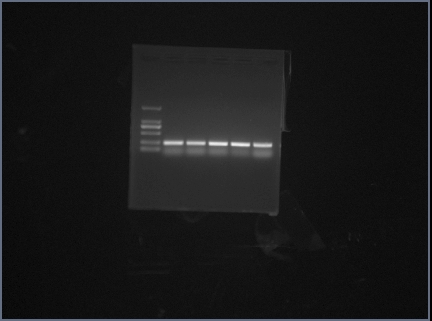

Supplement: Supplementary file 1 [file DataSheet1.ZIP › raw date/raw date/figure5/RT-PCR/b-actin.jpg]

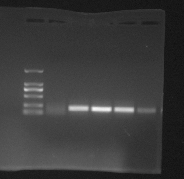

Supplement: Supplementary file 1 [file DataSheet1.ZIP › raw date/raw date/figure5/RT-PCR/ifn-γ.jpg]

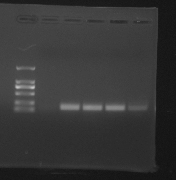

Supplement: Supplementary file 1 [file DataSheet1.ZIP › raw date/raw date/figure5/RT-PCR/il-17.jpg]

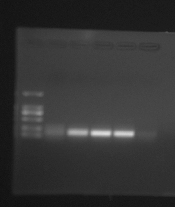

Supplement: Supplementary file 1 [file DataSheet1.ZIP › raw date/raw date/figure5/RT-PCR/il-2.jpg]

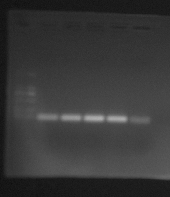

Supplement: Supplementary file 1 [file DataSheet1.ZIP › raw date/raw date/figure5/RT-PCR/tnf-α.jpg]

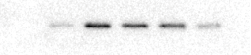

Supplement: Supplementary file 1 [file DataSheet1.ZIP › raw date/raw date/figure6/akt.png]

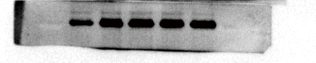

Supplement: Supplementary file 1 [file DataSheet1.ZIP › raw date/raw date/figure6/b-actin.png]

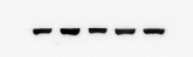

Supplement: Supplementary file 1 [file DataSheet1.ZIP › raw date/raw date/figure6/b-actin2.png]

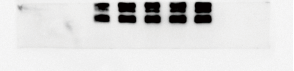

Supplement: Supplementary file 1 [file DataSheet1.ZIP › raw date/raw date/figure6/erk.png]

Figure 6A


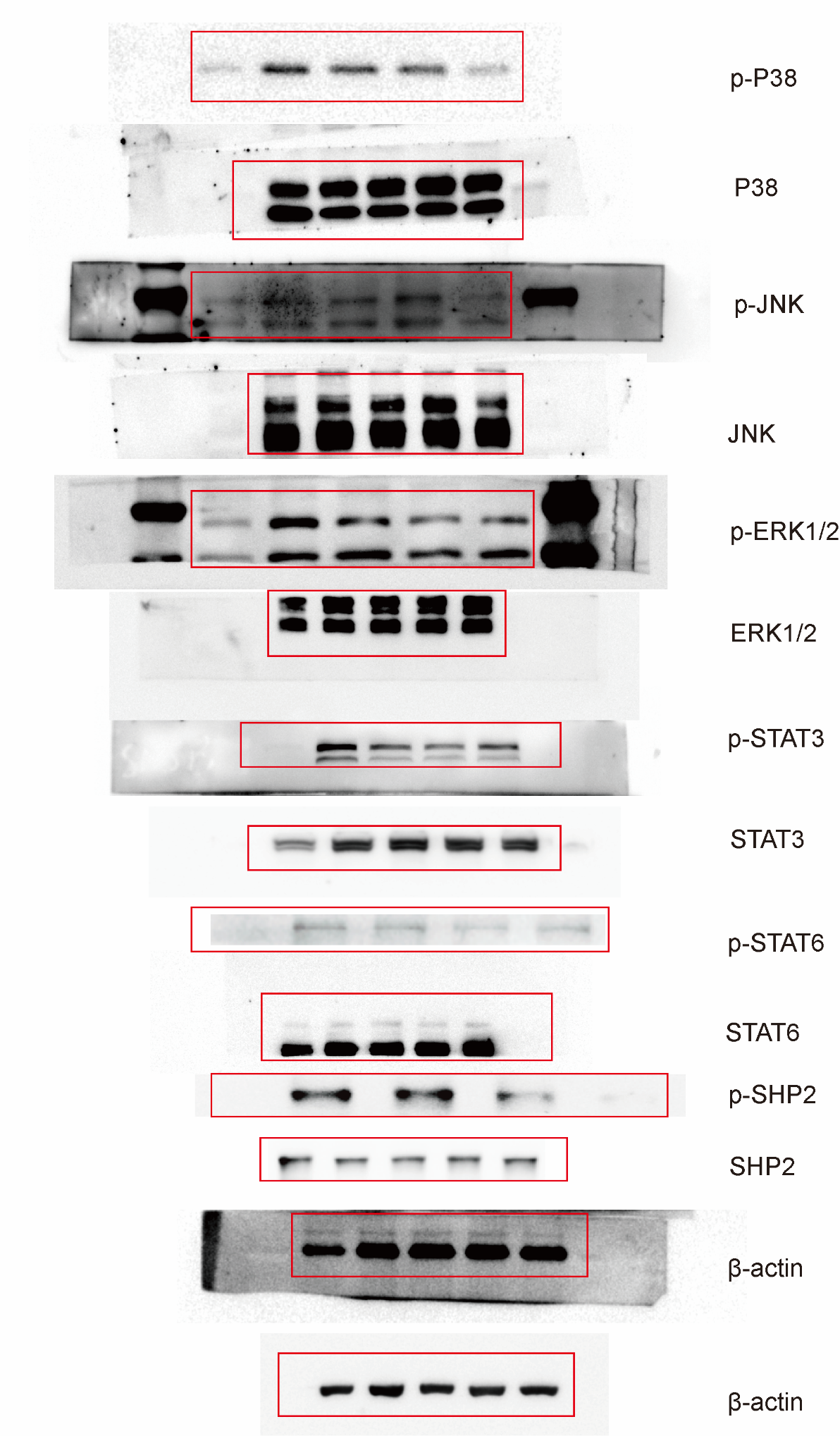

Supplement: Supplementary file 1 [file DataSheet1.ZIP › raw date/raw date/figure6/Figure 6A.docx]

Figure 6A

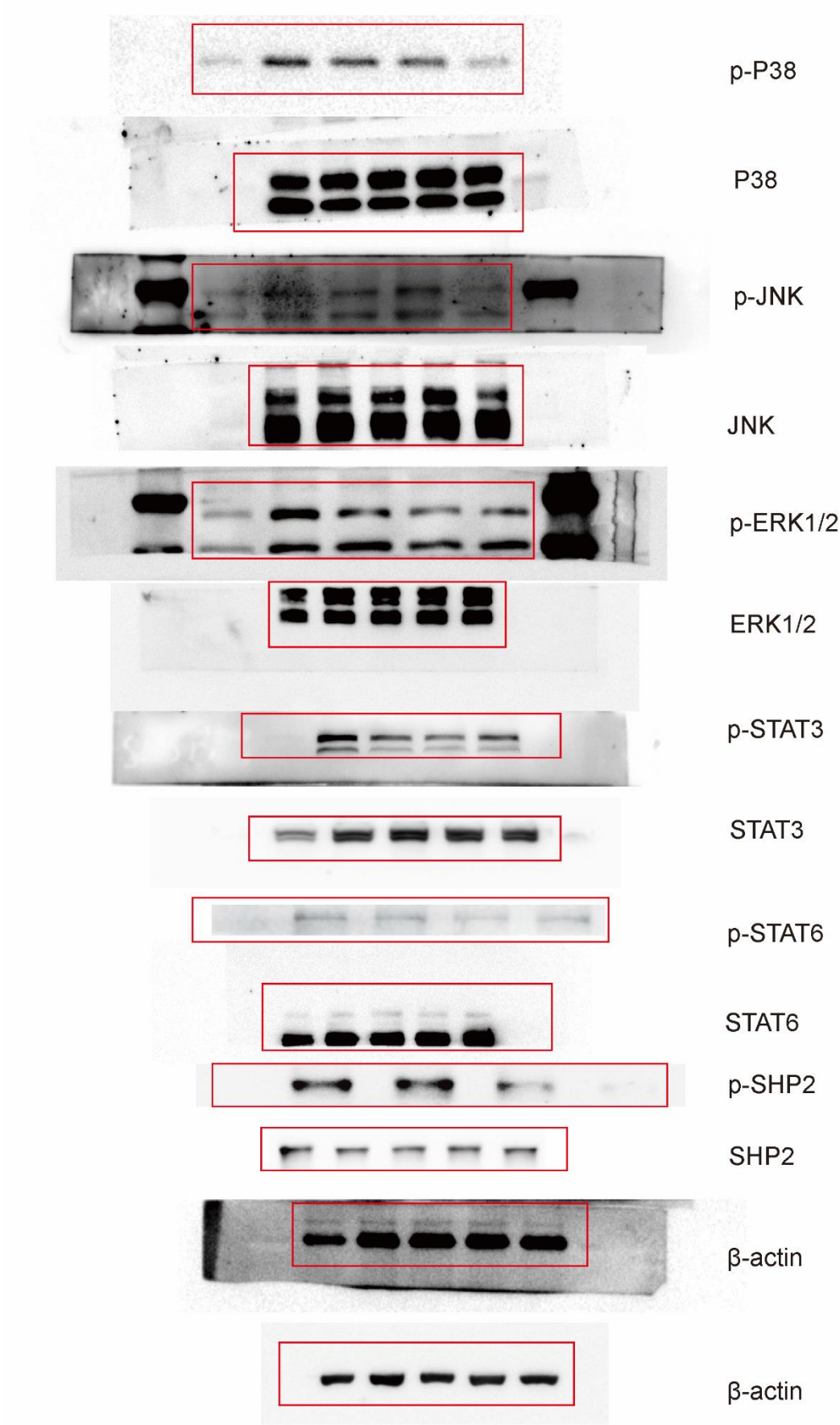

Supplement: Supplementary file 1 [file DataSheet1.ZIP › raw date/raw date/figure6/Figure 6A.pdf]

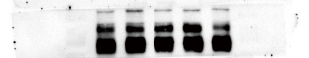

Supplement: Supplementary file 1 [file DataSheet1.ZIP › raw date/raw date/figure6/jnk.png]

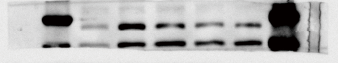

Supplement: Supplementary file 1 [file DataSheet1.ZIP › raw date/raw date/figure6/p-erk.png]

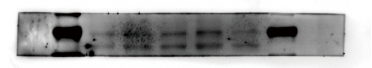

Supplement: Supplementary file 1 [file DataSheet1.ZIP › raw date/raw date/figure6/p-jnk.png]

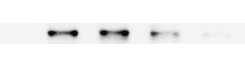

Supplement: Supplementary file 1 [file DataSheet1.ZIP › raw date/raw date/figure6/p-shp2.png]

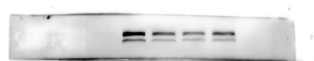

Supplement: Supplementary file 1 [file DataSheet1.ZIP › raw date/raw date/figure6/p-stat3.png]

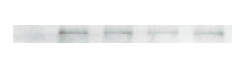

Supplement: Supplementary file 1 [file DataSheet1.ZIP › raw date/raw date/figure6/p-stat6.png]

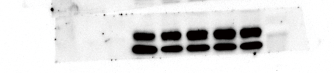

Supplement: Supplementary file 1 [file DataSheet1.ZIP › raw date/raw date/figure6/p38.png]

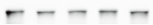

Supplement: Supplementary file 1 [file DataSheet1.ZIP › raw date/raw date/figure6/shp2.png]

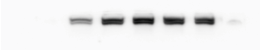

Supplement: Supplementary file 1 [file DataSheet1.ZIP › raw date/raw date/figure6/stat3.png]

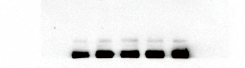

Supplement: Supplementary file 1 [file DataSheet1.ZIP › raw date/raw date/figure6/stat6.png]

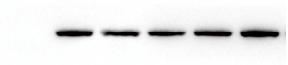

Supplement: Supplementary file 1 [file DataSheet1.ZIP › raw date/raw date/figure7/Figure 7C/b-actin.png]

Figure 7C


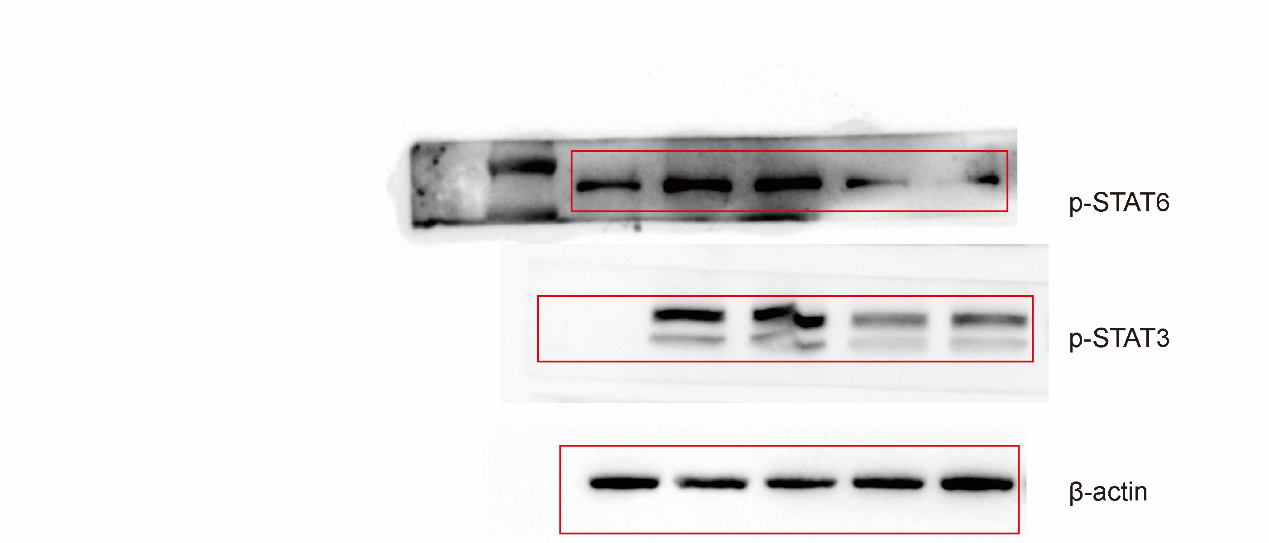

Supplement: Supplementary file 1 [file DataSheet1.ZIP › raw date/raw date/figure7/Figure 7C/figure 7C.docx]

Figure 7C

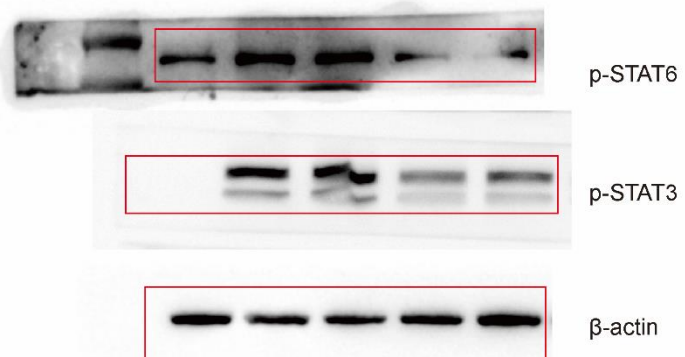

Supplement: Supplementary file 1 [file DataSheet1.ZIP › raw date/raw date/figure7/Figure 7C/figure 7C.pdf]

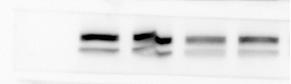

Supplement: Supplementary file 1 [file DataSheet1.ZIP › raw date/raw date/figure7/Figure 7C/stat3.png]

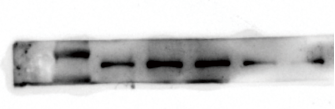

Supplement: Supplementary file 1 [file DataSheet1.ZIP › raw date/raw date/figure7/Figure 7C/stat6.png]

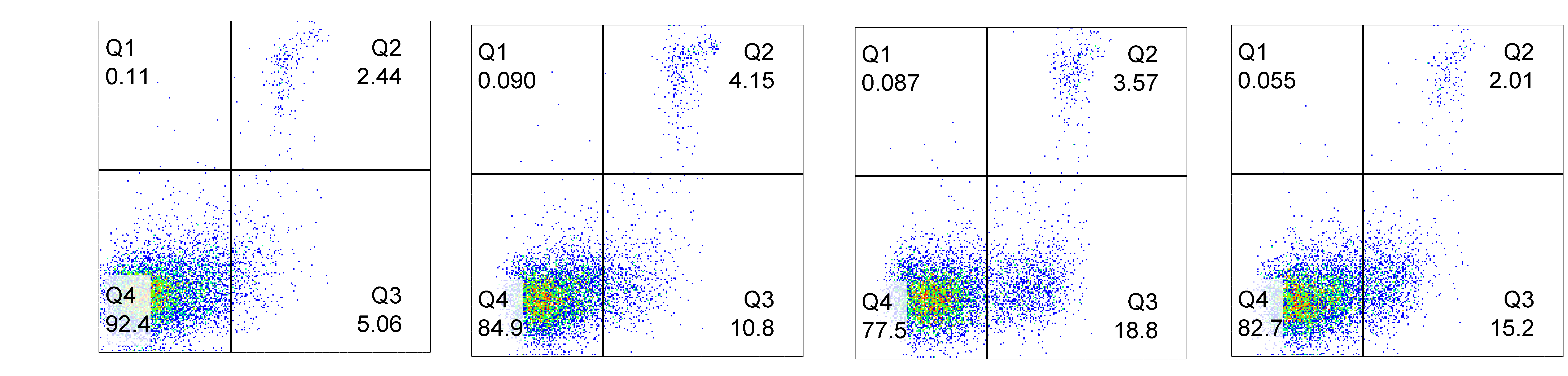

Supplement: Supplementary file 1 [file DataSheet1.ZIP › raw date/raw date/figure7/flow/flow.png]
